# SMYD5 is a ribosomal methyltransferase that catalyzes RPL40 lysine methylation to enhance translation output and promote hepatocellular carcinoma

**DOI:** 10.1038/s41422-024-01013-3

**Published:** 2024-08-05

**Authors:** Bisi Miao, Ling Ge, Chenxi He, Xinghao Wang, Jibo Wu, Xiang Li, Kun Chen, Jinkai Wan, Shenghui Xing, Lingnan Ren, Zhennan Shi, Shengnan Liu, Yajun Hu, Jiajia Chen, Yanyan Yu, Lijian Feng, Natasha M. Flores, Zhihui Liang, Xinyi Xu, Ruoxin Wang, Jian Zhou, Jia Fan, Bin Xiang, En Li, Yuanhui Mao, Jingdong Cheng, Kehao Zhao, Pawel K. Mazur, Jiabin Cai, Fei Lan

**Affiliations:** 1 Shanghai Key Laboratory of Medical Epigenetics, International Co-laboratory of Medical Epigenetics and Metabolism, Ministry of Science and Technology, Institutes of Biomedical Sciences, Key Laboratory of Carcinogenesis and Cancer Invasion, Ministry of Education, Liver Cancer Institute, Zhongshan Hospital, Fudan University, Shanghai, China; 2 China Novartis Institutes for BioMedical Research, Shanghai, China; 3 Department of Experimental Radiation Oncology, The University of Texas MD Anderson Cancer Center, Houston, TX USA; 4 Minhang Hospital & Institutes of Biomedical Sciences, Shanghai Key Laboratory of Medical Epigenetics, International Co-laboratory of Medical Epigenetics and Metabolism, Fudan University, Shanghai, China; 5 Department of Neurology of The Second Affiliated Hospital & Liangzhu Laboratory, Zhejiang University School of Medicine, Hangzhou, Zhejiang China

**Keywords:** Methylation, Ribosome, Liver cancer

## Abstract

While lysine methylation is well-known for regulating gene expression transcriptionally, its implications in translation have been largely uncharted. Trimethylation at lysine 22 (K22me3) on RPL40, a core ribosomal protein located in the GTPase activation center, was first reported 27 years ago. Yet, its methyltransferase and role in translation remain unexplored. Here, we report that SMYD5 has robust in vitro activity toward RPL40 K22 and primarily catalyzes RPL40 K22me3 in cells. The loss of SMYD5 and RPL40 K22me3 leads to reduced translation output and disturbed elongation as evidenced by increased ribosome collisions. SMYD5 and RPL40 K22me3 are upregulated in hepatocellular carcinoma (HCC) and negatively correlated with patient prognosis. Depleting SMYD5 renders HCC cells hypersensitive to mTOR inhibition in both 2D and 3D cultures. Additionally, the loss of SMYD5 markedly inhibits HCC development and growth in both genetically engineered mouse and patient-derived xenograft (PDX) models, with the inhibitory effect in the PDX model further enhanced by concurrent mTOR suppression. Our findings reveal a novel role of the SMYD5 and RPL40 K22me3 axis in translation elongation and highlight the therapeutic potential of targeting SMYD5 in HCC, particularly with concurrent mTOR inhibition. This work also conceptually broadens the understanding of lysine methylation, extending its significance from transcriptional regulation to translational control.

## Introduction

Protein lysine N^ε^-methylation plays a crucial role in various biological processes. While its impact on transcription regulation via histone proteins has been extensively studied over the past two decades or so, its role in translation remains largely unexplored. In this context, several mammalian ribosomal proteins, such as RPL4, RPL29, RPL40, and RPL36A, have been reported to contain lysine methylation.^1–3^ Among these, RPL40 is a special ribosomal protein encoded by the *UBA52* gene. The precursor UBA52 protein is a fusion protein of 128 amino acids (aa), comprising an N-terminal fusion of a ubiquitin module (76 aa). After the removal of ubiquitin, the mature form of RPL40 is 52 aa in length and is one of the last components assembled into the 60S ribosomal subunit in cytoplasm.^4,5^ In the mature 80S ribosome, RPL40 is located near the P stalk/GTPase Activation Center (GAC) and Sarcin-Ricin Loop (SRL), where elongation factors eEF1A and eEF2 bind. The elongation factors are crucial for recruiting peptidyl-tRNA to the A-site and for translocating it from A-site to P-site. RPL40 has been proposed to selectively regulate stress-related mRNA translation and confer resistance to elongation inhibitor Sordarin in yeasts,^4,6^ suggesting an essential function of RPL40 in protein synthesis. Importantly, the trimethylation of K22 on RPL40 (RPL40 K22me3, equivalent to UBA52 K98me3) was identified by mass spectrometry analysis in rat liver 27 years ago,^1^ and visualized in recent high-resolution ribosome structural studies.^2,3^ However, the role of this modification in translation and ribosome function remains unclear.

SET and MYND domain-containing (SMYD) proteins constitute an evolutionarily conserved subfamily of lysine methyltransferases, characterized by a catalytic SET domain split by a MYND domain.^7,8^ Among them, SMYD5 was recently reported to catalyze methylation of viral Tat protein and be involved in HIV infection,^9^ as well as to catalyze histone H3K36me3 at promoters and drive tumorigenesis in hepatocellular carcinoma (HCC),^10^ though the downstream mechanism is unclear. Indeed, data from the TCGA database indicate that *SMYD5* mRNA levels are elevated in most cancer types with HCC being one of the most significant types (Supplementary information, Fig. S1a).^11,12^ Consistently, two recent multi-omics studies have found that both *SMYD5* mRNA and protein levels are significantly elevated in HCC samples and are associated with poor clinical outcomes (Supplementary information, Fig. S1b–e).^13,14^

In this study, we identify SMYD5 as a ribosomal lysine methyltransferase that predominantly catalyzes RPL40 K22me3. The SMYD5-RPL40 K22me3 axis is crucial for efficient translation elongation and overall protein synthesis. Deficiency of SMYD5 in HCC cancer cells leads to hypersensitivity to mTOR inhibitors, likely due to a compounded inhibitory effect on protein synthesis. Employing both ex vivo and in vivo HCC models, we further elucidate the critical role of SMYD5-mediated RPL40 K22me3 in sustaining cancer growth, especially under suppressed mTOR signaling. These findings underscore the potential of targeting SMYD5-RPL40 K22me3 axis as a therapeutic strategy for HCC patients.

## Results

### SMYD5 trimethylates ribosomal protein RPL40 in vitro

**Fig. 1:**
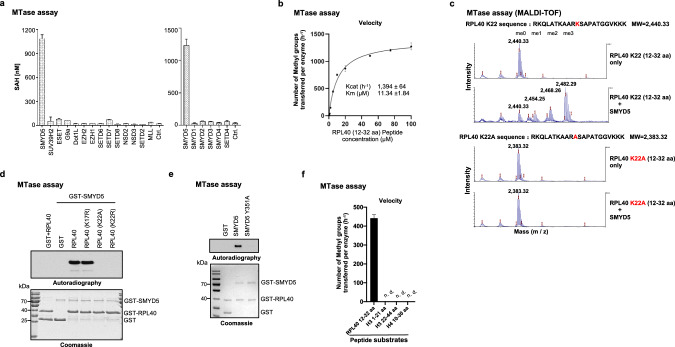
SMYD5 trimethylates ribosomal protein RPL40 in vitro*.* **a** In vitro methylation assays using recombinant RPL40 as substrate with a panel of indicated methyltransferases, with no enzyme as control (Ctrl.). The SAH converted by methyltransferase reactions from SAM was monitored by MTase-Glo Kit. All data are represented as mean ± standard deviation (SD) from 3 biological replicates. Two-tailed unpaired *t*-test. **b** Measurement of the enzymatic parameters, *Kcat* and *Km*, using 20 nM purified recombinant SMYD5 protein and the indicated concentrations of RPL40 peptide substrate (12–32 aa) for 30 min reactions. The measurements were performed by MALDI-TOF. **c** MALDI-TOF analyses of the methyltransferase activities of the recombinant SMYD5 on RPL40 wild-type (WT) and K22A mutant peptides (12–32 aa). The concentrations of peptides in reactions were all at 5 μM and the concentration of recombinant SMYD5 protein was at 20 nM. **d** In vitro SMYD5 methylation assays with the indicated recombinant RPL40 WT and mutant proteins, and GST protein (as control). Top panel, autoradiogram of the methylation assays; bottom panel, coomassie blue staining of the protein components in the reactions. **e** In vitro methylation assays using recombinant RPL40 as substrate with the GST-tagged WT and catalytic-dead (Y351A) SMYD5 proteins, and GST protein alone was used as a control. Top panel, autoradiogram of the methylation assays; bottom panel, coomassie blue staining of the protein components used in the reactions. **f** In vitro measurements of SMYD5 methyltransferase activities with the indicated substrates. RPL40 peptide was compared to histone tail-derived peptides. All peptides were at a concentration of 5 μM and reacted with a concentration of 20 nM purified recombinant SMYD5 protein for 30 min. The measurements were performed by MALDI-TOF.

To search for RPL40 methyltransferase, we screened a panel of SET domain-containing lysine methyltransferases for activity against the purified recombinant RPL40 protein. Using in vitro methyltransferase assay, we found that only SMYD5 methylated RPL40 (Fig. 1a). Using RPL40 peptide substrate (12–32 aa) and MALDI-TOF analyses, the *Km* and *Kcat* values were determined at 11.34 μM and 1394 h^−1^, respectively (Fig. 1b), with the value of *Kcat*/*Km* at ~122.9, demonstrating a strong enzymatic activity compared to other known lysine methyltransferases, such as G9A.^15^ We then confirmed that SMYD5 catalyzes trimethylation at the K22 site in both RPL40 recombinant protein and peptide (Fig. 1c; Supplementary information, Fig. S2a, b). The K22A replacement completely ablated the SMYD5 activity toward the peptide substrate (Fig. 1c). Further mutagenesis approaches using recombinant RPL40 protein as a substrate consistently demonstrated that SMYD5 methylation of RPL40 is specific to K22, as the K22A or K22R mutations ablated SMYD5 activity whereas K17R mutation did not (Fig. 1d). To exclude the possibility of co-purified contaminant associated with SMYD5 from *Escherichia coli*, we confirmed that the predicted catalytically dead SMYD5 mutant (Y351A, based on homology to SMYD2, SMYD3, and G9A), could not methylate recombinant RPL40 (Fig. 1e). In contrast to previous reports suggesting that SMYD5 is a putative histone methyltransferase,^10,16–19^ we have not detected any activity on mononucleosomes, histone H3 (1–21 aa, 22–44 aa), and H4 (10–30 aa) tail peptides (Fig. 1f; Supplementary information, Fig. S2c, d), while under the same condition, we could detect robust SMYD5 activity on RPL40 recombinant protein and peptide. Such results demonstrate that SMYD5 has strong in vitro methyltransferase activity toward RPL40 compared to histone substrates.

### RPL40 is the primary cytoplasmic substrate of SMYD5 in human cells

**Fig. 2:**
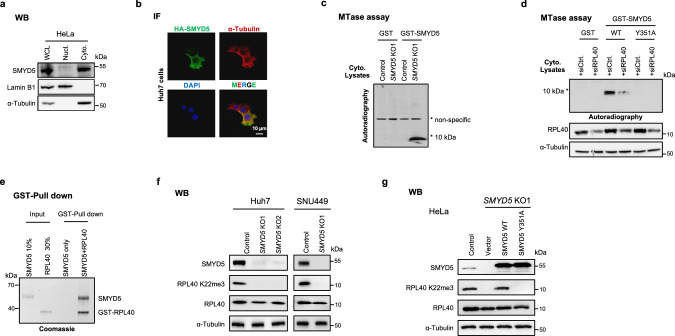
RPL40 is a primary cytoplasmic substrate of SMYD5 in human cells. **a** Western blot (WB) analysis of SMYD5 in the whole-cell lysate (WCL), cytoplasmic (Cyto), and nuclear fractions (Nucl) from HeLa cells. Lamin B1 and α-Tubulin were used as nuclear and cytoplasmic markers, respectively. **b** Immunofluorescence (IF) analyses of HA (green), α-Tubulin (red) and DAPI (blue) in Huh7 cells stably carrying HA-SMYD5 expression construct. **c** In vitro methylation reactions using recombinant GST-SMYD5 or GST (as a control) and cytoplasmic protein lysates from the control and *SMYD5* KO1 HeLa cells as substrates. * denotes the candidate substrate signal. **d** In vitro methylation reactions with recombinant GST-tagged WT, catalytic-dead SMYD5 (Y351A), or GST as control. Cytoplasmic protein lysates from control and RPL40 KD HeLa cells were used as substrates. Top panel, autoradiogram of the methylation assays; middle and bottom panel, WB analyses using the indicated antibodies. **e** In vitro pull-down assays using recombinant GST-tagged RPL40 and Flag-tagged recombinant SMYD5. **f** WB analyses of SMYD5, RPL40 K22me3 and RPL40 in the indicated cell lines. *SMYD5* KO1 and KO2 cell lines of Huh7 and *SMYD5* KO1 cell line of SNU449 were generated by corresponding gRNAs in Materials and Methods. **g** WB analyses of SMYD5, RPL40 and RPL40 K22me3 in cells. WT HeLa cells were used as the control. *SMYD5* KO1 HeLa cells were rescued by overexpressing empty vector, SMYD5 WT, and SMYD5 mutant (Y351A) constructs.

Congruous with our observation that SMYD5 lacks activity on histones, we observed that the endogenous and ectopically expressed SMYD5 predominantly localized to the cytoplasm in HeLa and Huh7 cells (Fig. 2a, b; Supplementary information, Fig. S3a, b). As RPL40 is known to be assembled to the 60S ribosomal subunit in the cytoplasm^4,5^ and shows predominantly cytoplasmic localization (Supplementary information, Fig. S3c, image available from Human Protein Atlas), we next tested whether RPL40 is the major cytoplasmic substrate of SMYD5. To investigate this in an unbiased fashion, we carried out a methyltransferase assay using total cytoplasmic protein lysates from either control or *SMYD5* knockout (KO) cells as substrates, and purified recombinant SMYD5 as an enzyme with ^3^H-SAM (S-adenosyl methionine) as the methyl donor. Strikingly, we found a single strong autoradiographic signal that was specifically associated with the addition of SMYD5, using the lysate from *SMYD5* KO cells (Fig. 2c). The signal migrated at molecular weight around and below 10 kDa, the predicted size of RPL40. Interestingly, we did not observe such a signal using cytoplasmic lysate from control HeLa cells under the same condition (Fig. 2c), indicating that the putative substrate might be close to being fully methylated in the control HeLa cells. Similar results were also observed in other human cell lines and mouse liver tissue (Supplementary information, Fig. S3d). We next performed tandem mass spectrometry (MS/MS) analyses of the gel-extracted HeLa proteins at ~10 kDa and extensively analyzed the data to search for, 1) peptide sequences with potential lysine methylation and 2) with protein MW ∼10 kDa. In this exercise, we filtered MS-derived peptides from 277 proteins, identifying only 6 hits that contained potential lysine methylation modifications. Notably, these hits included four ribosomal proteins, one of which was RPL40. These 6 peptides were synthesized and tested as substrates in an in vitro methylation assay with recombinant SMYD5 and ^3^H-SAM, and only the RPL40 peptide could be methylated (Supplementary information, Fig. S3e, f). Consistently, in vitro methylation assay using HeLa cytoplasmic lysate from cells depleted of RPL40 by RNAi as SMYD5 substrate resulted in a significant decrease in the ~10 kDa autoradiographic signal when incubated with WT but not the catalytically deficient recombinant SMYD5 (Fig. 2d). Altogether, these data indicate that RPL40 is the primary cytoplasmic substrate of SMYD5.

To further consolidate the finding, we performed Flag-SMYD5 immunoprecipitation (IP) followed by MS identification of SMYD5-interacting proteins. We found that the SMYD5 interactome contains many ribosome- and translation-related proteins (Supplementary information, Fig. S3g), with RPL40 scored as the second top binder. Although ribosomal proteins and translation factors are generally abundant and therefore often considered as contaminants in IP-MS experiments, the scores of unique peptides and peptide-spectrum match scores were of high confidence (> 55). The direct interaction between SMYD5 and RPL40 was also confirmed by GST pull-down assay (Fig. 2e).

To demonstrate whether SMYD5 is responsible for generating RPL40 K22me3 in cells, we raised a specific antibody against RPL40 K22me3 and validated it by dot blot assay (Supplementary information, Fig. S3h and Table S1). Further validation was conducted using a HEK293T CRIPSR knock-in (KI) cell line in which lysine 22 of endogenous RPL40 was mutated to arginine (K22R), resulting in no detectable signal in RPL40 K22me3 immunoblotting (Supplementary information, Fig. S3i). Subsequently, we generated *SMYD5* KO cells using CRISPR-Cas9 in HeLa, Huh7, SNU449, and HepG2 cell lines. We found that the RPL40 K22me3 signal was fully dependent on SMYD5 in all tested cell lines (Fig. 2f; Supplementary information, Fig. S3j). Furthermore, ectopical expression of WT but not the Y351A mutant SMYD5, restored RPL40 K22me3 in *SMYD5* KO HeLa cells (Fig. 2g). We next found that the ratios of K22me3/RPL40 were largely constant across multiple human cell lines and mouse tissues (Supplementary information, Fig. S3k, l). Targeted MS/MS analyses found that RPL40 K22me3 was the major form in Huh7 cells and mouse livers (Supplementary information, Fig. S3m), consistent with a previous report in rat livers.^1^

Together, these results support our in vitro findings and argue that SMYD5 is the physiologic enzyme that generates endogenous RPL40 K22me3, and that the K22-methylated RPL40 is the major cellular form of RPL40 in the samples tested in this study.

### RPL40 K22me3 structural proximity to 28S rRNA in ribosomal GAC

To understand how SMYD5-RPL40 K22me3 may affect ribosome function, we analyzed the recently reported high-resolution cryo-EM structure of the human mature ribosome.^3^ As mentioned in the introduction, RPL40 is located near the P stalk/GAC and SRL, where eEF1A and eEF2 bind (Supplementary information, Fig. S4a). The N-terminal alpha-helix (3–16 aa) associates with RPL9, which stabilizes the SRL, while the C-terminal domain, including K22, is inserted into a deep pocket formed by 28S rRNA helices H42, H89, H91 and H97, which are located next to the P stalk (Supplementary information, Fig. S4b, middle). This part of RPL40 adopted a zinc finger structure with C20, C23, C34, and C39 chelating a zinc molecule (Supplementary information, Fig. S4b, right). In the reported human ribosome structure (PDB 8GLP), the methyl electron density could readily be observed^3^ (Supplementary information, Fig. S4b right). The tri-methylated K22 (epsilon N) is positioned in a cleft between H42 and H89 of the 28S rRNA, with the closest distance to bases C4412 of H42 and G1945 of H89 at ~3.6 Å and 3.6 Å (Supplementary information, Fig. S4b right), respectively, a distance where van der Waals force may exist. Although the K22 side chain does not interact with translation factors, the H42 and H89 helices are known to interact with eEF2, eRF1, and A-site tRNA. Thus, it is plausible that without the trimethylation, the interaction and distance between RPL40 and 28S rRNA would be altered. Since the P stalk is a highly dynamic region during elongation, RPL40 methylation status could influence the overall P stalk conformation, which may impact the binding and release of elongation factors eEF1A and eEF2, potentially affecting the efficiency of protein synthesis.

### SMYD5 and RPL40 K22me3 affect polysome profiles and promote global translation output

We next investigated the impact of SMYD5 and RPL40 K22me3 on ribosome function and translation. First, using polysome profiling (ribosome sucrose gradient profiles), three consistent patterns in *SMYD5* KO HeLa and Huh7 cells were observed compared to the control cells (Supplementary information, Fig. S4c, d): 1) 40S, 60S and 80S fractions are largely unaffected by SMYD5 deletion; 2) *SMYD5* KO cells showed less heavy polysomes (> 5 ribosomes); 3) half-mer formation was observed in the light polysome and 80S fractions in the *SMYD5* KO cells, indicating altered active ribosome dynamics (also see Discussion), similar to what has been reported in yeast with one copy deletion of the two *RPL40* genes.^4^ These results indicated that loss of SMYD5 affects active ribosome patterning, which may lead to reduced translation activity.

**Fig. 3:**
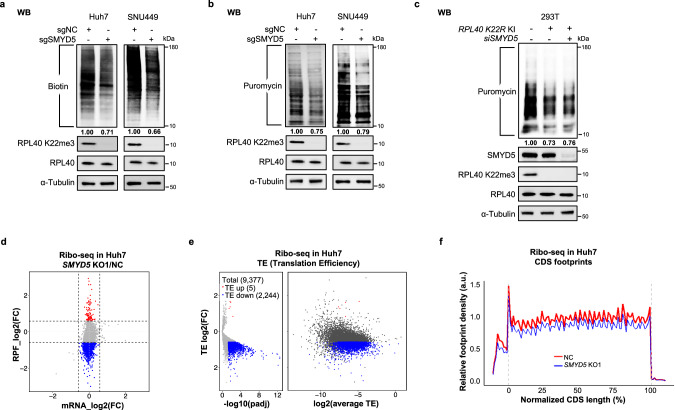
SMYD5 and RPL40 K22me3 promote global translation output by enhancing mRNA TE. **a**, **b** WB analyses of newly synthesized proteins in the negative control (NC) and *SMYD5*-depleted (KO1) Huh7 and SNU449 cell lines by AHA-click labeling (**a**) and puromycin (**b**) labeling approaches. The intensity of NC cells was normalized as 1.00 as indicated under the different treatments. **c** Measurement of newly synthesized proteins in the WT and *RPL40 K22R* KI 293T cell lines by SUnset/Puromycin labeling approach. **d** Scatterplots of ribo-seq depicting the changes in transcription level (*x*-axis) and translation level (*y*-axis). The red and blue dots represent genes with stable mRNA levels but increase or decrease in RPF levels. FC cutoff is 1.5. **e** Volcanoplots representing the TE changes from ribo-seq. Genes with FC > 1.5 or < 1/1.5 and *P*adj < 0.05 were colored. Average TE is between NC and *SMYD5* KO1, allowing for an evaluation of whether genes are highly/lowly translated. **f** Footprint of ribo-seq across normalized CDS regions, normalized by mitochondrial RPF.

We then evaluated the impact of SMYD5 and RPL40 K22me3 on translation output by measuring newly synthesized proteins in Huh7 and SNU449 HCC cell lines using two independent methodologies: AHA click chemistry and Puromycin labeling (SUnSET). Both methods consistently demonstrated a 20%–30% reduction in global translation output following SMYD5 deletion (Fig. 3a, b). A similar decrease in translation output was observed in the HEK293T *RPL40 K22R* KI cell line, connecting the effect to the RPL40 K22 site (Fig. 3c). Further knockdown of SMYD5 in HEK293T *K22R* KI cells did not exacerbate this defect, supporting the idea that SMYD5’s role in translation primarily functions through RPL40 K22me3 modification (Fig. 3c).

### SMYD5 enhances mRNA translation efficiency (TE)

To examine the SMYD5-dependent translational landscape, we then conducted ribosome profiling sequencing (ribo-seq) in Huh7 control and *SMYD5* KO cells.^20^ A significant decrease in ribosome-protected footprints (RPFs, i.e., ribosome protected fragments) was observed after *SMYD5* KO, indicating global reduced translation, while the transcriptome was largely unaffected (only few differentially expressed genes detected at fold change (FC) > 1.5) (Fig. 3d). We further calculated the TEs of reliably detected mRNAs (9377 transcripts), identifying a significant alteration in TEs among these transcripts (Fig. 3e; Supplementary information, Fig. S4e and Table S2). Specifically, 300 mRNAs exhibited significantly decreased TE (FC > 2, *P*adj (adjusted *P* value) < 0.05), and when the FC cutoff was adjusted to 1.5-fold, the number of mRNAs with decreased TE expanded to 2244, while those with increased TE remained comparatively fewer (4 by 2-fold cutoff and 5 by 1.5-fold cutoff), indicating a broad impact of SMYD5 loss on the translatome. We also noticed that most mRNAs with significant TE reduction had a relatively higher initial TE level (Fig. 3e, right). Additionally, ribosome footprint intensity tracks over coding DNA sequences (CDSs) showed a global reduction in ribosome binding in *SMYD5* KO cells, without preferential changes in distribution (Fig. 3f). Moreover, no differential codon occupancy was detected between control and *SMYD5* KO cells (Supplementary information, Fig. S4f), suggesting that the translation reduction is globally uniform.

These results collectively confirm that the SMYD5-RPL40 K22me3 axis plays a crucial role in maintaining robust global translation output.

### Loss of SMYD5-RPL40 K22me3 axis leads to elongation perturbation and hypersensitivity to translation inhibitors targeting A-site

**Fig. 4:**
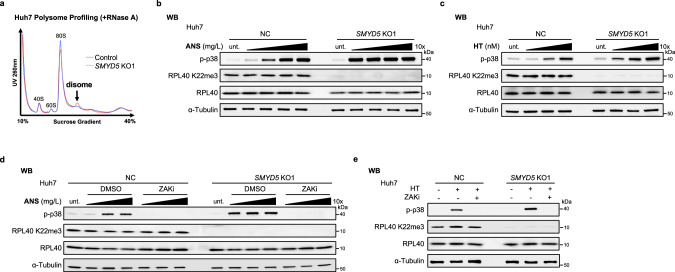
Loss of SMYD5-RPL40 K22me3 axis leads to elongation perturbation and hypersensitivity to translation inhibitors targeting A-site. **a** Polysome profiles from lysates with RNase A treatment of NC and *SMYD5* KO1 Huh7 cell lines. Black arrows denote the disomes. **b**, **c** WB analyses for phosphorylation of p38 in the NC and *SMYD5* KO1 Huh7 cell lines treated with ANS (0.001–1 mg/L, 15 min) or Harringtonine (HT) (0.1–10 μM, 15 min). **d**, **e** WB analyses for phosphorylation of p38 in the NC and *SMYD5* KO1 Huh7 cell lines treated with ANS (0.01–1 mg/L, 15 min) or HT(10 μM, 15 min). Before ANS or HT treatment, the NC and *SMYD5* KO1 Huh7 cell lines were treated with DMSO or ZAK inhibitor M443 (5 μM, 1 h) as indicated. Note: α-Tubulin was used as a control for panels in all above and below WB analyses.

As mentioned earlier, RPL40 is located near the GAC, a region where elongation factors bind and is closely related to the A-site function (Supplementary information, Fig. S4a). The proximity leads us to hypothesize that the loss of SMYD5 and RPL40 K22me3 could disturb elongation. A common indicator of elongation perturbation is ribosome stalling and collision, frequently resulting in disome formation.^21,22^ Consistent with our hypothesis, increased disome fractions were observed in Huh7 and SNU449 cell lines following SMYD5 depletion (Fig. 4a; Supplementary information, Fig. S5a). A similar effect was also observed in HEK293T *RPL40 K22R* KI cells (Supplementary information, Fig. S5b).

Recent studies have shown that significant ribosome stalling and collisions activate the ribosome-associated kinase ZAK, which in turn promotes p38 phosphorylation,^22,23^ a key ribotoxic stress response (RSR) pathway. Although depletion of SMYD5 and RPL40 K22me3 alone was insufficient to activate p38, as indicated in Fig. 4b–e; Supplentary information, S5c–f (comparing the untreated condition), we speculated that the elongation perturbations caused by SMYD5 and RPL40 K22me3 loss might result in elevated sensitivities to RSR inducers. To test this idea, we measured the dosage-dependent p38 activation of several commonly used RSR inducers, including Anisomycin (ANS, targeting A-site), Harringtonine (HT, targeting A-site), Cycloheximide (CHX, targeting E-site), 254 nm UV irradiation (UVB) and Menadione (an inducer of Reactive Oxygen Species, ROS).^3,22–24^ We found that *SMYD5* KO Huh7 cells exhibited an ~100-fold greater sensitivity to ANS and an ~10-fold greater sensitivity to HT in activating p38 compared to control cells (Fig. 4b, c). Notably, this activation of p38 was entirely dependent on ZAK (Fig. 4d, e).^22,23^ Similarly, HEK293T *K22R* KI cells also demonstrated increased sensitivity to ANS treatments, directly linking the effect to the RPL40 K22 (Supplementary information, Fig. S5c).

Interestingly, SMYD5 loss in Huh7 cells did not significantly alter sensitivities to CHX, UVB, and Menadione (Supplementary information, Fig. S5d–f), in contrast to the A-site targeting inhibitors, ANS and HT. These findings suggest that SMYD5-RPL40 K22me3 depletion specifically impairs ribosome fitness upon elongation perturbation and sensitizes elongating ribosomes to low levels of A-site targeting inhibitors. Such observed SMYD5-dependent elongation effects align with the physical positioning of RPL40 K22me3 near the GAC, which plays a crucial role in elongation and A-site function.

### SMYD5 loss sensitizes cancer cells to mTOR pathway blockade

Elevated protein synthesis and translation activity are hallmarks of cancer,^25^ and as mentioned earlier, SMYD5 is frequently overexpressed in HCC and associated with poor clinical outcomes (Supplementary information, Fig. S1). We therefore postulated that SMYD5-mediated RPL40 K22me3 may promote HCC tumorigenesis. To explore this idea, we focused our investigation on SMYD5 function in three HCC cancer cell lines (Huh7, SNU449, and HepG2) and one model cell line HeLa.

**Fig. 5:**
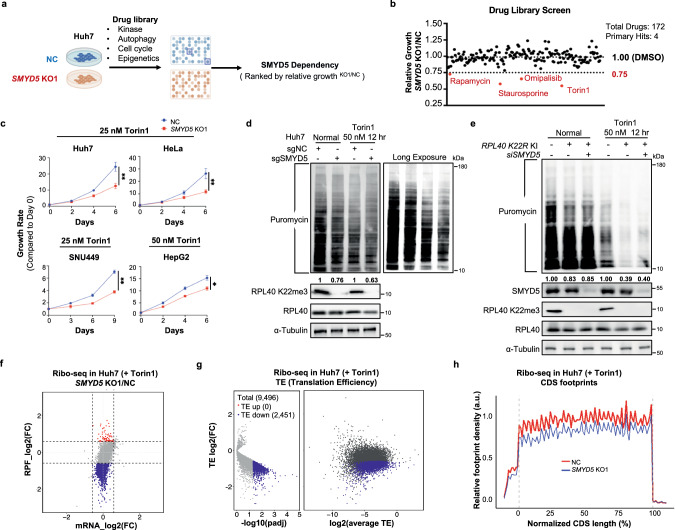
SMYD5 deletion sensitizes cancer cells to mTOR blockade. **a** Schematic of drug screen in the control and *SMYD5* KO1 Huh7 cells. **b** Scatter plot of the fitness of *SMYD5* KO1 Huh7 cells compared to the NC Huh7 cells under the treatment of individual drugs for 4 days. Data presented as the relative growth rate of *SMYD5* KO1/NC cells. Four hits caused more than 25% growth retardation of *SMYD5* KO cells compared to the control cells were in red. Data were presented as the mean from duplicated experiments. **c** Proliferation analyses of the control and *SMYD5*-depleted (KO1) Huh7, HeLa, SNU449 and HepG2 cells under the treatment of Torin1 at the indicated concentrations. Experiments were performed three times. **d** WB analyses of newly synthesized proteins in the NC and *SMYD5* KO1 Huh7 cell lines with or without Torin1 treatment by puromycin labeling approach. **e** WB analyses of newly synthesized proteins in the WT, *RPL40 K22R* KI, and *RPL40 K22R* KI with further knockdown of SMYD5 HEK293T cell lines with or without Torin1 treatment by puromycin labeling approach. **f** Scatterplots of ribo-seq depicting the changes between control and KO cells in transcription level (*x*-axis) and translation level (*y*-axis) with 50 nM Torin1 treatment for 12 hr. The red and blue dots represent genes with stable mRNA levels but increase or decrease in RPF levels. FC cutoff is 1.5. **g** Volcanoplots of ribo-seq representing the TE changes. Genes with FC > 1.5 or < 1/1.5 *P* adj < 0.05 were colored. Average TE is between NC and *SMYD5* KO1 cells, allowing for an evaluation of whether genes are highly/lowly translated. **h** Footprint of ribo-seq across normalized CDS regions under Torin1 treatment, normalized by mitochondrial RPF.

Although SMYD5 depletion did not impact the proliferation of all four cell lines (Supplementary information, Fig. S6a) in normal cell culture conditions, we explored potential connections between SMYD5 and other pathways. To this end, we performed a comparative drug screen in control and *SMYD5* KO Huh7 cells using a library consisting of 172 small-molecule inhibitors of major signaling, growth, and epigenetics pathways (Fig. 5a; Supplementary information, Table S3). Interestingly, we found that the 4 top hits causing reduced fitness of *SMYD5* KO cells compared to the control cells, were inhibitors of the mTOR pathway, including Rapamycin, Torin1, Omipalisib (dual mTOR/PI3K inhibitor), and an apoptosis inducer Staurosporine that suppresses 4EBP1 phosphorylation (Fig. 5b). Such results raise the possibility that SMYD5 loss enhances mTOR dependency in Huh7 cells. Accordingly, viability assays showed elevated sensitivity of Huh7 cells to mTOR inhibitors, Torin1 and Rapamycin, upon SMYD5 loss, with IC_50_ values shifted roughly 5 times lower (Supplementary information, Fig. S6b). Consistently, rescue experiment demonstrated that K22R mutation was deficient in restoring Huh7 growth potential under Torin1 treatment as compared to the WT RPL40 (Supplementary information, Fig. S6c). Elevated sensitivity to mTOR inhibition upon SMYD5 loss was also observed in SNU449, HepG2, and HeLa cells (Fig. 5c; Supplementary information, Fig. S6d), indicating a broader phenomenon.

To assess the impact of SMYD5 loss on translation under mTOR suppression, we employed AHA click chemistry and SUnSET labeling techniques. Both methods confirmed a more pronounced SMYD5-dependent reduction in newly synthesized proteins in Huh7 and SNU449 cell lines under mTOR suppression compared to baseline conditions (Fig. 5d; Supplementary information, Fig. S6e–g; compare the ratios of reduction, lane 4/lane 3 vs lane 2/lane 1). Consistently, an enhanced decrease in newly synthesized proteins was observed in 293T *RPL40 K22R* KI cell lines under Torin1 treatment (Fig. 5e, compare the ratios of reduction, lane 5/lane 4 vs lane 2/lane 1). Further knockdown of SMYD5 in *RPL40 K22R* KI cells did not enlarge this defect, connecting the SMYD5-dependent effect to RPL40 K22 (Fig. 5e, compare the ratios of reduction, lane 6/lane 4 vs lane 5 /lane 4). Additionally, ribo-seq analysis in Huh7 cells identified a significant number (491 out of a reliably detected total of 9496, FC > 2,  *P*adj < 0.05 ) of mRNAs with reduced TEs upon SMYD5 loss under Torin1 treatment, while no mRNAs showed significantly increased TEs. When the cutoff was lowered to FC 1.5, 2451 mRNAs showed reduced TEs while still, no mRNAs showed significantly increased TE (Fig. 5f, g; Supplementary information, Fig. S6h and Table S2). In addition, similarly as shown in Fig. 3f, a greater global reduction of ribosome binding was observed over the CDS regions under Torin1 treatment in *SMYD5* KO cells (Fig. 5h). Again, no differential codon occupancy was detected under Torin1 treatment (Supplementary information, Fig. S6i), same as the untreated normal condition (Supplementary information, Fig. S4f).

Gene set enrichment analysis (GSEA) of ribo-seq reads revealed that G2M checkpoint, MYC targets, E2F targets, and ribosome biogenesis were enriched in control cells compared to *SMYD5* KO upon Torin1 treatment (Supplementary information, Fig. S6j–l), in line with the growth defect of *SMYD5* KO cells under this condition (Fig. 5c). Consistent with these, under 12 h of Torin1 treatment, both Huh7 and HeLa *SMYD5* KO cells showed fewer 40S, 60S, 80S and heavy polysome abundance compared to the control cells (Supplementary information, Fig. S6m). WB analyses also showed a moderate reduction in a few core ribosomal proteins in the *SMYD5* KO Huh7 cells upon mTOR suppression (Supplementary information, Fig. S6n), suggesting that the combined effect of SMYD5 loss and mTOR suppression might influence ribosome biogenesis, partly explaining the reduced growth potential of SMYD5-depleted cells under mTOR suppression. Taken together, these findings demonstrate that *SMYD5* KO cells experience substantial translation suppression when treated with Torin1.

### Loss of SMYD5 and mTOR inhibition synergize to suppress HCC development

**Fig. 6:**
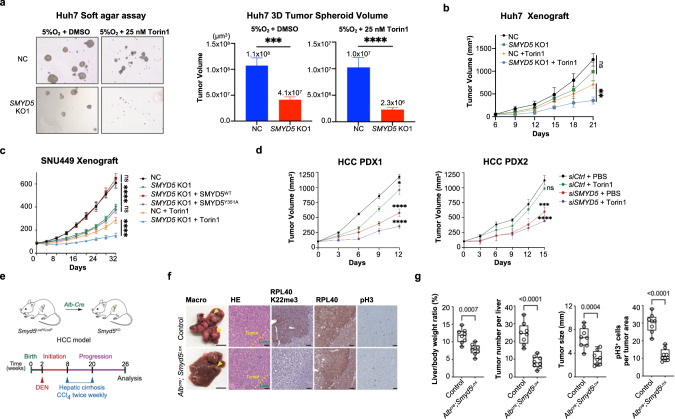
SMYD5 promotes HCC both ex vivo and in vivo. **a** Left, anchorage-independent growth analyses using 3D soft agar assay of the control and *SMYD5* KO1 Huh7 cells under indicated treatments; quantification of the 3D spheroid volumes of the results was shown in the right panel. **b** Tumor volume quantification for Huh7 xenografts in nude mice (*n* = 4 for each group). The control and *SMYD5* KO1 Huh7 xenografts were both treated with PBS or Torin1 25 mg/kg daily. Data were represented as mean ± SEM. **c** Tumor volume quantification for SNU499 xenografts in NSG mice (*n* = 5 for each group). The control and *SMYD5* KO1 SNU449 cells, and the *SMYD5* KO1 SNU449 cells overexpressing WT or catalytically deficient (mut) SMYD5 were used. 25 mg/kg Torin1 was used for intraperitoneal injection daily for the indicated groups. Data were represented as mean ± SEM. **d** Tumor volume quantification of HCC PDX in NSG mice (*n* = 4 for each group) from different two HCC patients. The siRNA that dissolved in the PBS with DMSO or 500 nM Torin1 was used for intratumoral injection every three days. Data were represented as mean ± SEM. **e** Schematic illustrating the animal HCC model utilized in the generation of liver-specific Smyd5 deletion; Lower, experimental design to assess effects of SMYD5 ablation on development of HCC with advanced liver fibrosis. **f** Left two columns, representative gross images of liver pathology (arrows indicate tumor nodules). HE-stained sections and IHC staining with the indicated antibodies of tumors from control and SMYD5-depleted mice at 6 months of age (representative of *n* = 8 mice for each group). Scale bars: 5 mm (whole mount) and 100 µm (section). **g** Quantifications of liver/body weight ratio, tumor number, tumor size, and pH3 positive cells in control and SMYD5-depleted tumor samples used in Fig. 6f. Boxes: 25th to 75th percentile; whiskers: min to max; center line: median; *n* = 8 mice for each group.

To further explore SMYD5 function in HCC tumorigenesis when mTOR is suppressed, we monitored Huh7 cell proliferation using 3D soft agar and xenograft assays. We performed soft agar assay under 5% physioxia oxygen condition,^26^ and found that the *SMYD5* KO Huh7 cells showed moderate defects in growth compared to the control cells, and the difference in growth potential was further pronounced upon mTOR inhibition (Fig. 6a). Similar results were observed in vivo, where 25 mg/kg Torin1 treatment led to significantly reduced xenograft growth of Huh7 *SMYD5* KO cells compared to the control Huh7 cells (Fig. 6b), while no statistical difference in xenograft growth was observed without mTOR inhibition (Fig. 6b). The Huh7 xenograft results were further supported by experiments using the human SNU449 cell line, which was established from poorly differentiated HCC. However, different from Huh7, SMYD5 ablation alone could readily attenuate xenograft growth of SNU449, and the growth was restored by complementation with WT but not with catalytically inactive SMYD5 (Fig. 6c; Supplementary information, Fig. S7a). Importantly, the SMYD5 dependency of SNU499 xenograft tumor growth was further magnified by Torin1 treatment as well (Fig. 6c).

To establish the clinical significance of SMYD5 and RPL40 K22me3, we analyzed 202 human HCC patient samples from a Zhongshan Hospital cohort (Shanghai), performing IHC examinations to quantify the abundance of SMYD5, RPL40, and RPL40 K22me3. The analysis revealed predominant cytoplasmic staining of SMYD5, which was significantly elevated in 56% of tumor (T) samples and lower in only 6% of the tumor (T) samples compared to adjacent paratumor (P) tissues (Supplementary information, Fig. S7b, c). The intensity of SMYD5 staining inversely correlated with overall survival and disease-free survival rates (Supplementary information, Fig. S7d), consistent with previous reports (Supplementary information, Fig. S1).^13,14^ Like SMYD5, RPL40 K22me3 is elevated in 53.4% and attenuated in 5.0% of the tumor (T) samples compared to adjacent paratumor (P) tissues (Supplementary information, Fig. S7c). RPL40 also exhibited a similar, though less pronounced, trend (Supplementary information, Fig. S7c), consistent with the notion that the expression of ribosomal proteins is generally elevated in cancer.^27^ Further analysis revealed that RPL40 K22me3 not only correlated strongly with the RPL40 level but also with the SMYD5 level in these human HCC samples (Supplementary information, Fig. S7e, f). To further demonstrate clinical relevance, we then tested the SMYD5 effect in two HCC patient-derived xenograft (PDX) models. Using specially modified siRNAs for in vivo delivery (see Materials and Methods), we depleted SMYD5 and RPL40 K22me3 in HCC PDX tumors via intratumoral injections. The results demonstrated that treatment of *SMYD5* siRNA alone readily resulted in significantly suppressed HCC PDX growth, with an even greater effect achieved by combined treatment of Torin1 (Fig. 6d; Supplementary information, Fig. S7g). Altogether, these findings underscore the critical role of SMYD5 and RPL40 K22me3 in promoting HCC progression.

### SMYD5 promotes HCC development in vivo

HCC develops most often as a complication of liver cirrhosis. To investigate the role of SMYD5 in HCC tumorigenesis from the cirrhosis stage, we interbred conditional *Smyd5*^*loxP/loxP*^ mutant mice with hepatocyte-specific Cre-recombinase strain (*Alb*^*Cre*^), resulting in specific *Smyd5* gene deletion in the liver (Fig. 6e). Animals with liver-specific SMYD5 depletion (*Alb*^*Cre*^*;Smyd5*^*loxP/loxP*^) developed normally without any notable phenotype, aligning with data from International Mouse Phenotyping Consortium (IMPC, Supplementary information, Table S4) showing that full body *Smyd5* KO mice are viable, fertile and exhibit only minor phenotypes.^28,29^ We utilized a model based on a two-stage chemical application to initiate and promote hepatocellular tumors in association with advanced liver fibrosis. The HCC model was induced by a single injection of genotoxic *N*-nitrosodiethylamine (DEN) followed by repeated administration of the pro-fibrogenic agent carbon tetrachloride (CCl_4_) (Fig. 6e). The control animals (*Alb*^*Cre*^) developed advanced HCC at 6 months of age. By contrast, SMYD5 ablation led to a significant reduction of overall tumor sizes and tumor burden with fewer tumor nodules noted (Fig. 6f, g). The liver/body weight ratio, a common indicator of total HCC tumor burden, was also significantly lower in *Smyd5* KO animals (Fig. 6f). In addition, depletion of SMYD5 resulted in a complete loss of RPL40 K22me3 staining signal and attenuation of tumor cancer cell proliferation (pH3) (Fig. 6f, g), in support of our mechanistic study. These observations are also consistent with the postulated role of SMYD5 and RPL40 K22me3 in HCC tumorigenesis.

## Discussion

Despite RPL40 K22 methylation has been identified in rat liver in 1997,^1^ its role in ribosome function and translation control has not been explored due to the unawareness of its enzyme. Here, we identify SMYD5 as a ribosome protein methyltransferase responsible for RPL40 K22me3, allowing subsequent assessment of the role of this modification in translation. We demonstrate that the SMYD5 methylation on RPL40 K22 enhances global translation output and is required for proper translation elongation. Loss of SMYD5 and methylation on RPL40 leads to elevated ribosome collisions and hypersensitivity to translation inhibitors targeting A-site (Fig. 4a–c; Supplementary information, Fig. S5a–c). RPL40 is positioned near GAC where elongation factors, eEF1A and eEF2, bind. Conformational dynamics at this region is foreseeable during elongation, which involves cycles of A-site tRNA loading and A- to P-site transition. The increased sensitivities particularly to translation inhibitors targeting A-site indicate that RPL40 K22me3 is important in maintaining proper GAC conformation and dynamics, likely through the interaction of the methylated sidechain of K22 with 28S rRNA (Supplementary information, Fig. S4a, b). Of note, our ribo-seq analyses find that SMYD5 loss causes strong TE reduction of thousands of translating mRNA, without involving codon-specific effects (Fig. 3d, e; Supplementary information, Fig. S4e, f). Based on these findings, we conclude that the SMYD5-RPL40 K22me3 axis is required for general elongation but not selective translation. In addition, SMYD5 may potentially impact initiation and 60S assembly as indicated by observed half-mers (Supplementary information, Fig. S4c, d), which requires further investigation.

The observation of elevated ribosome collisions or stalling upon loss of SMYD5-RPL40 K22me3 is of interest (Fig. 4a; Supplementary information, Fig. S5a, b). Recently, ribosome collisions/stalling and subsequent activation of RSR pathway have been connected to a variety of upstream stresses and cellular processes, such as inflammation, aging, and liver metabolic disorder.^24,30^ Of note, interfering with a ribosome modifier/modification causing ribosome collisions has not been reported yet. Our findings suggest that the SMYD5 and RPL40 K22me3 axis enhances ribosome fitness under conditions of low-level elongation stress, particularly associated with A-site perturbation. Whether the axis is an integral part of the ribosome stress-sensing pathway is an intriguing question for future studies. As yeast ribosomes lack this modification (Supplementary information, Fig. S7h),^31^ and close SMYD5 homolog start to appear from insects. It is plausible that SMYD5 and RPL40 K22me3 have evolved in higher organisms for ribosomes to confront more complex environmental stresses. Through in-depth structural comparison using currently available structures with enough high resolutions (Supplementary information, Fig. S8), we have not observed significant alteration of RPL40 and its K22 side chain due to translation factor binding. Therefore, the detailed mechanism of how RPL40 K22me3 affects A-site function and GAC/P-stalk dynamics remains unclear and our findings encourage future investigation toward this direction.

Elevated ribosome biogenesis and translation output are hallmarks of cancer,^27^ and overexpression of ribosome proteins has been linked to enhanced cancer proliferation and metastasis.^32,33^ Recent studies have expanded the scope to include rRNA modifications in cancer,^34,35^ and some modifications exhibit variable levels between cancer and normal tissues, suggesting a mechanism for ribosome heterogeneity.^36^ Yet, the role of ribosome lysine methylations and their modifiers in cancer remains underexplored. Although our data suggest that RPL40 is close to fully methylated in human cell lines and mouse tissues tested (Supplementary information, Fig. S3d, k–m), future studies should thoroughly investigate the physiological/pathological ranges of RPL40 K22me3 and the regulation of SMYD5 itself. In this context, the observed elevation of SMYD5, RPL40, and RPL40 K22me3 in HCC, correlated with poor prognosis, underscoring the clinical relevance of our discoveries. HCC often arises from complex metabolic, epigenomic, and proteomic reprogramming, rather than from direct genetic mutations,^37^ making effective targeted therapies for HCC scarce. While mTOR inhibitors have shown limited efficacy in treating HCC,^38,39^ our findings demonstrate that disrupting SMYD5-mediated methylation of RPL40 K22me3 enhances the sensitivity of HCC cells to mTOR inhibition. This indicates that a dual-targeting approach could surpass the effectiveness of mTOR inhibition alone.

Our SNU449 xenograft and PDX HCC models demonstrate that SMYD5 depletion alone significantly suppresses cancer growth, and renders them hypersensitive to mTOR suppression (Fig. 6b, c). Furthermore, the genetically engineered mouse model of cirrhosis-associated HCC with hepatocyte-specific *Smyd5* KO indicated that the SMYD5-RPL40 K22me3 pathway is critical in tumor initiation and progression (Fig. 6e–g). Compared to its limited effect in cell culture assays (Supplementary information, Fig. S6a), the observed robust phenotype of SMYD5 ablation in animal models could be explained by the constrained mTOR activity in vivo, due to limited nutrition and oxygen availability.^40^ Our study thus nominates SMYD5 as a novel therapeutic target for HCC, whose efficacy could be further enhanced in combination with mTOR suppression. Of note, given that siRNA approaches have recently been shown to be effective for liver-related gene targeting in humans, our findings from HCC PDX models are of significant clinical value (Fig. 6d; Supplementary information, Fig. S7g). Importantly, since SMYD5 is also elevated in other cancer types (TCGA, Supplementary information, Fig. S1a), future research should focus on developing specific inhibitors or modulators of SMYD5 as a general approach for cancer therapy.

Targeting the translational machinery — a strategy that has proven successful in hematopoietic malignancies with the ribosome-targeting agent (Homoharringtonine, also called Omacetaxine) developed from Chinese medicine and been granted accelerated approval by FDA in 2012^41–43 ^— remains underexplored with ribosomal modifiers. Here, we find that targeting SMYD5 may exhibit favorable safety profiles, as evidenced by the normal liver development and viability of *Smyd5* KO mice (Supplementary information, Table S4). These suggest that targeting ribosome modifier SMYD5 might present fewer toxicity issues compared to direct targeting ribosomes.

In contrast to the well-established role of histone lysine methylation in transcription regulation, the function of lysine methylation in ribosomal proteins during translation remains largely unexplored. Similar to nucleosomes, ribosomes are ribonucleoprotein complexes made of positively charged small ribosome proteins, enriched with lysine and arginine and negatively charged rRNAs. Such physicochemical properties lay the foundation for analogous epigenetic mechanisms to exist and to directly influence ribosomes. Indeed, in addition to RPL40, previous studies have identified lysine methylation on mammalian RPL4, RPL29, and RPL36A.^1–3,44^ It is unclear whether lysine methylation occurs on more ribosomal proteins, particularly those located at flexible regulatory regions that current structure analyses cannot visualize. Our study thus calls for future research in this emerging area, aiming to expand the understanding of lysine methylation’s significance beyond transcription to encompass ribosome biology.

## Materials and methods

### Cell culture, antibodies, and general reagents

HeLa, HEK293T, Huh7, HepG2, and SNU449 cells were cultured in Dulbecco’s Modified Eagle’s Medium (DMEM, Hyclone) supplemented with 10% fetal bovine serum (FBS, Gibco). HeLa, HEK293T, Huh7, and HepG2 cell lines were obtained from the National Collection of Authenticated Cell Cultures (China), and SNU449 was obtained from ATCC.

For the RPL40 knockdown experiments, specific siRNA was synthesized (5′-CCUGCGAGGUGGCAUUAUU-3′) and introduced into the cells using RNAiMAX (Invitrogen) at ∼50% confluency. For the rescue experiment in Supplementary information Fig. S6c, the RPL40 coding sequence of CGCCTGCGAGGTGGCATTATT (WT) was modified to CGGCTCCGGGGAGGGATCATC (seven synonymous mutations) to create RNAi resistance. Cells were collected at 60–96 h post siRNA transfection for further analyses. For rescue experiments, the pLenti-EF1a-BSD vector (Addgene) system was utilized. Lentiviruses were made in 293T cells and the viral supernatants were collected at 60 h post-transfection and passed through a 0.45-μm filter prior to infection. After infection, HeLa and Huh7 cells were selected under 2 μg/mL Puromycin (Gibco) or 4 μg/mL Blasticidin (Gibco) for 5 days before cell proliferation analyses. For activating the phosphorylation of p38 and inhibiting ZAK in cells, anisomycin (ANS), Homoharringtonine (HHT), harringtonine (HT), Menadione (Mena), and M443 were obtained from MCE (HY-18982, HY-N0862, HY-14944, HY-B0332 and HY-112274).

Primary antibodies used in this study are listed in Supplementary information, Table S2.

### Generation of *SMYD5* KO cell lines and *RPL40 K22R* KI cell lines

CRISPR-Cas9 targeting system was utilized as previously described.^45^ Guide RNA sequences for *SMYD5* KO are: KO1: 5′-CTGAGCAATACCACCAGGTC-3′, and KO2: 5′-AGCGCGGGTCTCCGTGGAAG-3′. Both sequences were designed to target exon1. The gRNA sequence for *RPL40* KI is: 5′-CATCGGAGCACACATACTTG-3′ and the donor template is: 5′-GCCAGCTGGCCCAGAAATACAACTGCGACAAGATGATCTGTCGCAGGTATGTGTGCTCCGATGCTTGGGGGGCTGTGGGGGCTGCC-3′

### Cell fractionation separation

Cells were swelled in hypotonic buffer (10 mM HEPES, pH 7.5, 1.5 mM MgCl_2_, 10 mM KCl, 0.5 mM DTT, with Cocktail protease inhibitors (Roche) and 1 mM PMSF) and incubated on ice for 20 min, and then treated with 0.2% Triton X-100 for 5 min for lysis. Nuclei were collected by centrifugation at 2000 rpm for 10 min at 4 °C, and the supernatants were saved as cytoplasmic fraction.

### WB analysis

Cells were lysed by 2× SDS-PAGE loading buffer and boiled at 95 °C for 10 min. Tissue samples were firstly homogenized by tissue grinders in lysis buffer containing 150 mM NaCl, 25 mM TrisCl, 1% Triton X-100, 1% sodium deoxycholate, 0.1% SDS, protease inhibitor (Roche) and phosphatase inhibitors (MedChemExpress) and then boiled at 95 °C for 10 min with 5× SDS-PAGE loading buffer. Before loading for vertical SDS-PAGE gel electrophoresis, all samples needed to perform centrifugation at 12,000 rpm and collect the supernatant. After performing electrophoresis, proteins in the samples would transfer from the gel to the nitrocellulose membrane. The membrane was blocked by 5% skim milk with TBST after transfer and then incubated with the specific antibodies. Finally, the membrane was incubated in the chemiluminescent substrate solution and western blot signals were detected using the ChemiDoc imaging system (Biorad).

### IF and IP

Cells were seeded in 24-well plates at a density of 5 × 10^4^ cells/well at 24 h before IF examination. Cytosol extracts used for IP and co-IP were prepared from HeLa cells and the experiments were carried out as described previously.^46^

### GST pull-down assay

Coding sequences of *SMYD5* and *RPL40* were cloned into pGEX4T-1 and GST-tagged recombinant proteins were purified using affinity resins from SMART Lifesciences (Changzhou, China). Flag-tagged SMYD5 and other recombinant methyltransferases were purchased from Active Motif China Inc. (Shanghai).

A total of 2.5 μg Flag-SMYD5 and 1 μg GST-RPL40 recombinant proteins were mixed and incubated with 150 μL binding buffer (20 mM TrisCl 7.4, 150 mM NaCl, and 0.2% Triton X-100). Protein complex of GST-RPL40 and Flag-SMYD5 was immobilized by 10 μL GST resin and Flag-SMYD5 alone was set up as control. The resin and protein complexes were then washed with binding buffer for 4 times and subsequently subjected to SDS-PAGE examination and coomassie blue staining.

### Methyltransferase assay

Recombinant GST-SMYD5, Flag-SMYD5, and other indicated methyltransferases (Fig. 1a) were used as candidate enzymes. Recombinant GST-RPL40, RPL40 derived peptides (12–32 aa), histone tail-derived peptides, mononucleosomes, and the indicated cytosolic protein lysates were used as substrates. In general, 10 nM to 200 nM of individual methyltransferases were used and 5–10 μM substrates were used. For Fig. 2c and d, 20 μg of cytosolic protein lysates were incubated with 100 nM recombinant SMYD5. Methyltransferase buffer contained 20 mM TrisCl 8.0, 0.02% Triton X-100, and 0.5 mM TCEP (tris (2-carboxyethyl) phosphine) with 10 μM SAM (or ^3^H-SAM), and assays were generally performed in a 10–20 μL reaction system, for 2 h unless indicated.

The methyltransferase activities were monitored by four approaches, i.e., MALDI (Fig. 1b, c, f; Supplementary information, Fig. S2b, c), MS/MS after propionylation as previously described^47^ (Supplementary information, Fig. S2a), SDS-PAGE separation followed by radioautography (Figs. 1d, e, 2c, d; Supplementary information, Fig. S3f), and MTase-Glo Kit (Promega, Fig. 1a; Supplementary information, Fig. S2d). *Kcat* and *Km* analyses were performed by fitting Michaelis–Meanten equation with Graphpad. RPL40 peptides were synthesized from Qiangyao Biotech Co., Ltd. (Shanghai), and histone peptides were purchased from Active Motif China Inc. (Shanghai).

### Sample preparation for targeted MS and parallel reaction monitoring (PRM) data acquisition

To establish and optimize the PRM method, we methylated the RPL40 (1–35 aa) peptide in vitro by recombinant SMYD5. The methylation process was validated by MALDI-TOF. The unmethylated peptide segment was protected through propionylation and the peptide underwent reduction alkylation and trypsin digestion. Total protein samples from cells and tissues were lysed in SDS loading for SDS-PAGE gel electrophoresis. Approximately 100 μg total proteins of each sample were separated by electrophoresis. The RPL40-containing fractions were carefully excised from the gel at a molecular weight of ~10 kDa. The gel was then propionylated to protect unmodified residues, followed by in-gel reduction alkylation using 40 mM CAA (chloroacetamide) and 10 mM TCEP. In-gel digestion was performed using Trypsin (2 ng/μL) for 6–8 h. Digested peptides were extracted using a solution of 50% acetonitrile and 0.1% trifluoroacetic acid (TFA). Then peptides were desalted using a C18 column and subsequently freeze-dried. Sample peptides were analyzed using on-line nanospray LC-MS/MS on an UltiMate 3000 system (ThermoFisher Scientific, MA, USA) coupled to a timsTOF Pro mass spectrometer (Bruker Daltonics).

### MS analysis

The data analysis was performed using SpectroDive 11.10 with default parameters. The software automatically corrected retention times and mass windows and determined the optimal extraction window automatically. Peptide identification was conducted with a confidence threshold of Q value ≤ 0.01.

### Cell proliferation and chemical library screen

For growth analyses of HeLa, Huh7, and HepG2 cells, a total of 1 × 10^5^ cells per well were seeded into 12-well plates, the viabilities were measured at the indicated time point by CellTiter-Lumi^TM^ kit (Beyotime Inc.). Cell viability was measured every 2 or 3 days and the cells were split to ∼20% density per well to ensure continuous growth at the logarithmic phase. For the chemical library screen, a total of 1500 control and *SMYD5* KO1 Huh7 cells per well were seeded in 96-well plates, cells with compound treatment were cultured for 4 days, and then subjected to the measurement of enhanced Cell-Counting Kit-8 activity (Beyotime Inc.). Detailed information of individual compounds and concentrations used in the screen can be found in Supplementary information, Table S2.

### Rescue experiment

For the RPL40 rescue experiment in Huh7 cells, we used siRNA to knockdown endogenous RPL40 and restored RPL40 expression by transiently transfecting siRNA-resistant version of WT and K22R constructs simultaneously. Growth analyses were initiated 48 h after the first transfection (set as Day 0). The second transfection was conducted in another 48 h (Day 2 in the growth analyses) to sustain the knockdown of endogenous RPL40 and the ectopic expression of WT and K22R versions of RPL40.

### Soft agar assay

A total of 500 cells per well were diluted in the top layer containing 2.5% low viscosity methyl cellulose with complete medium (Life Technologies), and cultured on a bottom layer of 1% agar with complete medium in 12-well dishes. Both top and bottom layer medium were supplemented with 1% Penicillin-Streptomycin (Gibco) and 10% FBS. Additional media were added every 7 days to keep humidity before counting at day 25. The tumor volumes were collected and analysed by ImageJ (version 1.53).

### Xenograft assay

For Huh7 xenograft assay, four-week-old female athymic nu/nu mice (BALB/c) housed under specific pathogen-free conditions were used in this study. A total of 2 × 10^6^ Huh7 (double check) cells were injected into the mammary fat pads of mice at the density of 2 × 10^6^ cells/mL. Mice were sacrificed and tumors were excised at day 18 after injection. Tumor development was measured every two days. Torin1 treatment and data collection were conducted as below. The animal care and experimental protocols were carried out in accordance with procedures and guidelines established by Shanghai Medical Experimental Animal Care Commission, all animal experiments were approved by Fudan University Institutional Committee.

For SNC449 xenograft assay, cells transduced with lentivirus expressing sgRNA/Cas9 and indicated reconstitution vectors expressing WT or mutant SMYD5 were transduced to immunocompromised 8-week-old NSG mice (*NOD.SCID-IL2Rg*^*–*^*/*^*–*^). The transplantation was performed by subcutaneous injection of cells mixed with matrigel (1:1) 2 × 10^6^ cells to the flanks of mice. When tumors became palpable, they were calipered every 4 days to monitor growth kinetics. Mice were treated as indicated with Torin1 (25 mg/kg once per day, IP) in the vehicle 40% (2-hydroxypropyl)-β-cyclodextrin. Control animals underwent the same procedure but received vehicle treatment. All animals were numbered, and experiments were conducted in a blinded fashion. After data collection, treatment groups were revealed, and animals assigned to groups for analysis. Tumor size was measured using a digital caliper and tumor volume was calculated using the formula: Volume = (*width*)^2^ × *length*/2 where *length* represents the largest tumor diameter and *width* represents the perpendicular tumor diameter. The endpoint was defined as the time at which a progressively growing tumor reached 20 mm in its longest dimension as approved by the MDACC IACUC protocol (00001636, PI: Mazur), and in no experiment was this limit exceeded.

### PDX assay and siRNA delivery in vivo

PDXs were obtained from the Liver Cancer Institute, Zhongshan Hospital, Fudan University, Shanghai, China, and ethical approval (B2022-573R) was obtained from the research ethics committee of Fudan University affiliated Zhongshan Hospital, and written informed consent was obtained from each patient. PDX models were established by transplanting small tumor fragments quickly and directly from surgical specimens of specific HCC patients into the flanks subcutaneous tissues of NSG mice. Excess tumor tissue can be frozen for the next inoculation. To knockdown the level of SMYD5 and RPL40 K22me3 in PDX tumors in situ, we delivered siRNA modified by cholesterol, phosphorothioate (PS), 2′-*O*-methyl (2′-OMe), and 2′-deoxy-2′-fluoro (2′-F) into tumors by intratumoral injection per three days from the beginning of the measurement. Measurements were performed every two days when tumors were macroscopic. Tumor volume was calculated using the formula: Volume = (*width*)^2^ × *length*/2 where length represents the largest tumor diameter and width represents the perpendicular tumor diameter.

### HCC animal model and IHC

Reporter-tagged insertion with conditional potential *Smyd5*^*tm1a(EUCOMM)*^ mouse strain was obtained from the European Mouse Mutant Archive repository.^29^ Founder mice were crossed with *Rosa26*^*FlpO*^ deleter strain^48^ to generate conditional *Smyd5*^*LoxP/LoxP*^ allele.

*Alb*^*Cre*^ mice have been described before^49^ and were obtained from the Jackson Laboratory (003574). Mice were maintained on a mixed C57BL/6;129S1 strain background and we systematically used littermates as controls in all the experiments. For liver-specific deletion of SMYD5 we interbreed *Alb*^*Cre*^ and *Smyd5*^*LoxP/LoxP*^ mice. To establish HCC mouse model, a single dose of 1 mg/kg DEN (N-Nitrosodiethylamine, Sigma-Aldrich) was intraperitoneally injected (i.p.) into male mice at 2 weeks of age, followed by repeated administration of a low dose of a pro-fibrogenic agent carbon tetrachloride (CCl_4_, Sigma-Aldrich) at 0.2 mL/kg i.p. two times per week starting from 8 weeks of age for 12 weeks.^50^ Six weeks after the last injection, mice were sacrificed for macroscopic and histopathological liver examination. All animals were numbered, and experiments were conducted in a blinded fashion. After data collection, genotypes were revealed, and animals were assigned to groups for analysis. All mice were co-housed with littermates (2–5 per cage) in a pathogen-free facility with standard controlled temperature of 22 °C, with a humidity of 30%–70%, and a light cycle of 12 h on/12 h off set from 7 am to 7 pm and with unrestricted access to standard food and water under the supervision of veterinarians, in an AALAC-accredited animal facility at the University of Texas M.D. Anderson Cancer Center (MDACC). Mouse handling and care followed the NIH Guide for Care and Use of Laboratory Animals. All animal procedures followed the guidelines of and were approved by the MDACC Institutional Animal Care and Use Committee (IACUC protocol 00001636, PI: Mazur).

Tissue specimens were fixed in 4% buffered formalin for 24 h and stored in 70% ethanol until paraffin embedding. 3 μm sections were stained with hematoxylin and eosin (HE) or used for immunostaining studies. IHC was performed on formalin-fixed, paraffin-embedded tissue (FFPE) sections using a biotin-avidin HRP conjugate method (Vectastain ABC kit). After incubated with 1st antibodies, sections were developed with DAB and counterstained with hematoxylin. Pictures were taken using a PreciPoint M8 microscope equipped with the PointView software.

### Polysome profiling

Polysome profiling was prepared as previously described,^51^ with modifications. Approximately 2 × 10^7^ Huh7 and HeLa cells were incubated with 400 μM CHX (MCE) for 10 min and then pelleted. Pellets were washed twice in PBS with 400 μM CHX and immediately lysed in 200 µL cold lysis buffer (100 mM KCl, 10 mM MgCl_2_, 50 mM TrisCl, pH 7.4 and 0.5% NP-40) for 10 min on ice and pipetted to homogenize. The lysates were clarified by centrifugation at 700× *g* for 5 min at 4 °C to discard the cell nucleus and 12,000× *g* for 10 min at 4 °C to discard mitochondria and debris. Lysates were then loaded onto 10%–50% sucrose gradients and ultracentrifuged in an SW41 Ti swinging-bucket rotor (331362, Beckman) at 36,000 rpm for 2 h at 4 °C. For RNase A digested polysome profiling,^22^ lysates containing 100 μg of total RNA were treated with RNase A (ThermoFisher Scientific) at 5 mg/L for 45 min at room temperature and added 400U of RNase Inhibitor (Beyotime Inc.) to terminated the digest reaction. Digested lysates were then loaded onto 10%–40% sucrose gradients and ultracentrifuged in an SW41 Ti swinging-bucket rotor (331362, Beckman) at 36,000 rpm for 2 h at 4 °C. Samples were fractionated using a Biocomp gradient fractionator for absorbance polysome profiles and separation. Control and *SMYD5* KO samples were measured and loaded evenly with equivalent 260 nM OD values.

### Ribo-seq and data processing

Ribo-seq was performed as previously described,^52^ and ARTseq Ribosome Profiling Kit’s instructions with modifications. About 10^7^ cells were pre-treated with 100 μg/mL CHX (MCE) for 10 min at 37 °C, then washed and collected by ice-cold PBS containing 100 μg/mL CHX. Cells were lysed by 120 μL Mammalian Polysome Buffer (10 mM Tris-HCl, pH 7.5, 100 mM KCl, 5 mM MgCl_2_, 1% Triton X-100 with 1× protease inhibitor cocktail (Roche)) for 10 min. After centrifugation at 13,000× *g* for 10 min at 4 °C. 10 μL lysate was kept for mRNA-seq and purified by RNA Clean & Concentrator-5 kit (RCC-5 R1016, Zymo), followed by rRNA removal with Ribo-off rRNA Depletion (N406, Vazyme) and library preparation with VAHTS Universal RNA-seq Library Prep Kit (NR605, Vazyme). Add about 30U RNase I (EN0601, ThermoFisher Scientific) to 100 μL lysate and digest for 45 min at room temperature. Digestion was stopped by adding 4 μL of Superase-In (AM2696, ThermoFisher Scientific). Meanwhile, MicroSpin S-400 HR columns (27514001, Cytiva) were equilibrated with 3 mL of Mammalian Polysome Buffer by gravity flow and emptied by centrifugation at 600× *g* for 4 min. We then immediately loaded 100 μL of the digested lysate on the column and eluted the column by centrifugation at 600× *g* for 2 min. RNA was extracted by RCC-5 and separated on 15% denaturing urea-PAGE gel. After SYBR gold (S11494, ThermoFisher Scientific) staining, the size ranges from 25–40 nt was cut out and recovered by a small-RNA PAGE Recovery Kit (R1070, Zymo). The eluted RNA was mix with Superase-In, T4 PNK, and Buf A (EK0031, ThermoFisher Scientific) at 37 °C for 15 min and supplemented by 1 mM ATP (R0441, ThermoFisher Scientific) for another 30 min before extraction by RCC-5 and generation library by Small RNA Library Prep Kit (NR811, Vazyme). The libraries were sequenced by NovaSeq 6000 (conducted by Nanjing Gaoxin Precision Medicine Technology Co., Ltd.).

After sequencing, reads were trimmed by TrimGalore v0.6.10 to excise low-quality bases and adapters in both mRNA and Ribo-seq datasets. Quality assessment was subsequently conducted utilizing FastQC. Contamination originating from ribosomal RNA was discerned through alignment of reads to human rRNA sequences employing Bowtie2^53^ v2.3.5.1, followed by the exclusion of mapped reads from subsequent analyses. The human genome reference sequence (GRCh38.p14.genome.fa) and annotation files (gencode.v45.chr_patch_hapl_scaff.annotation.gtf) were acquired from the GENCODE browser. Within the annotation file, the longest isoforms were retained using AGAT v1.2.1 (agat_sp_keep_longest_isoform.pl), and only the canonical chromosomes (1–22, X, Y) were selected in the sequence file. The remaining Ribo-seq reads were aligned to the filtered human reference genome using STAR^54^ v2.7.10b with specified parameters (—runMode alignReads—readFilesCommand zcat—quantMode TranscriptomeSAM GeneCounts—twopassMode Basic). RNA-seq clean reads were analyzed using a combination of HISAT2^55^ v2.2.1 and StringTie^56^ v2.1.7. Raw mRNA counts were normalized using DESeq2^57^ with exclusive retention of highly expressed genes (Average FPKM in NC and KO > 2.5). RPF reads were normalized relative to the cumulative counts of mitochondrial RPFs^58^ TE was determined by dividing RPF counts by mRNA counts. Statistical analysis of differentially transcribed genes within each sample pair was executed using the R package Xtail.^59^ Codon bias for each group was computed utilizing CONCUR^60^ v1.0, while codon occupancy was quantified and depicted through customized Python scripts. Read density of focused transcripts was calculated employing RiboMiner^61^ v0.2 and visualized using custom Python scripts. GSEA analysis was performed by GSEA software v4.3.3 based on hallmark gene sets and ribosome biogenesis gene set.^62,63^

### HCC tissue microarray and IHC

A total of 202 formalin-fixed paraffin-embedded HCC tissues (containing tumor and paratumor compartments) were collected from consecutive patients with HCC who underwent curative resection from 2006 to 2007 at the Liver Cancer Institute of Fudan University (Shanghai, China). Histopathological diagnoses were based on World Health Organization criteria. Ethical approval was obtained from the research ethics committee of Fudan University affiliated Zhongshan Hospital, and written informed consent was obtained from each patient. Among the 202 patients, 172 cases were male, 30 cases were female. The age distribution was from 27 to 84 years old. Tumor size ranged from 0.5 cm to 20 cm. Follow-up data were summarized at the end of December 2013, with a median follow-up of 51 months (range: 5–73 months). Tissue microarrays were constructed and IHC was performed as previously described elsewhere.^64^

### Statistical analysis

For cell proliferation assays, all statistical data were calculated using GraphPad Prism 9. Comparisons of data were performed by two-sided, parametric, and unpaired *t*-test; *P* values of less than 0.05 were considered significant. *n* = 3 or 4 (the number of samples) for each experimental group. Values are presented as mean ± SD. Three biological replicates were performed and one representative was shown.

The statistical data were calculated using GraphPad Prism 9 for soft agar assays. Data comparisons were performed by two-sided, parametric, and unpaired *t*-tests; *P* values of less than 0.05 were considered significant. *n* = 3 (the number of samples) for each experimental group. Values are presented as mean ± SD. 3 biological replicates were performed and one representative was shown.

For IHC experiments, statistical analysis was performed with SPSS software (19.0; SPSS, Inc., Chicago, IL). Values are presented as mean ± SD. The Student’s *t*-test was used for comparisons between groups. Pearson’s correlation analyses were performed for the IHC intensities of SMYD5, RPL40, RPL40 K22me3, and HIF1A. Overall survival rates were analyzed using Kaplan–Meier’s method and the log-rank test. *P* < 0.05 was considered statistically significant.

### Supplementary information

Supplementary information, Fig S1

Supplementary information, Fig S2

Supplementary information, Fig S3

Supplementary information, Fig S4

Supplementary information, Fig S5

Supplementary information, Fig S6

Supplementary information, Fig S7

Supplementary information, Fig S8

Supplementary information, Figure Legends

Supplementary information, Table S1

Supplementary information, Table S2

Supplementary information, Table S3

Supplementary information, Table S4

## Data Availability

High-throughput sequencing data were deposited to the Gene Expression Omnibus with an accession number GSE241588.
