# Supplementary figures and images for: SMYD5 is a ribosomal methyltransferase that catalyzes RPL40 lysine methylation to enhance translation output and promote hepatocellular carcinoma
Source: Cell Res. 2024 Aug 5;34(9):648–60. doi: 10.1038/s41422-024-01013-3 (PMC11369092; doi:10.1038/s41422-024-01013-3)

Fig. S1

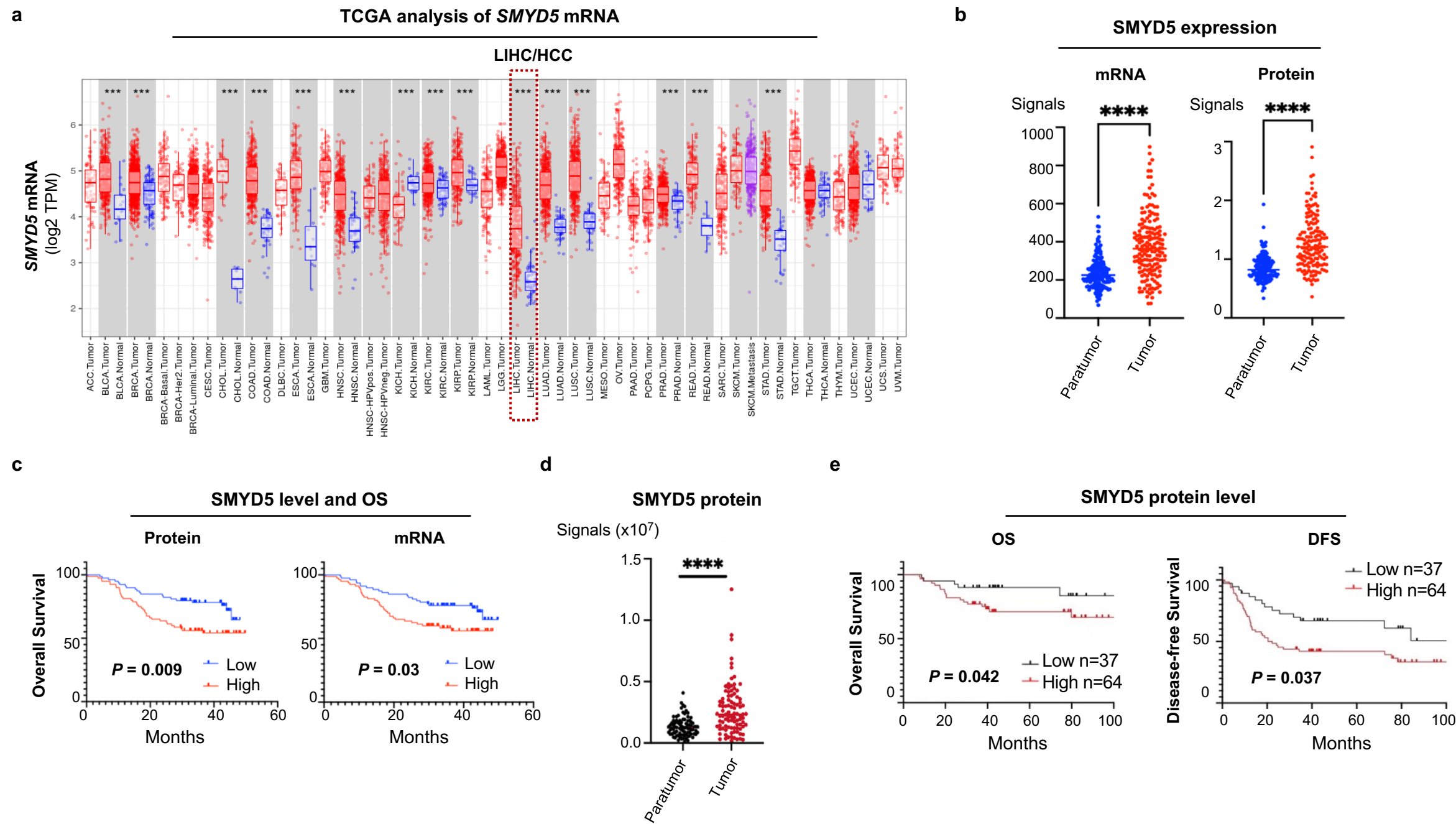

Supplement: Supplementary file 1 — Supplementary information, Fig S1 [file 41422_2024_1013_MOESM1_ESM.pdf]

Fig. S3

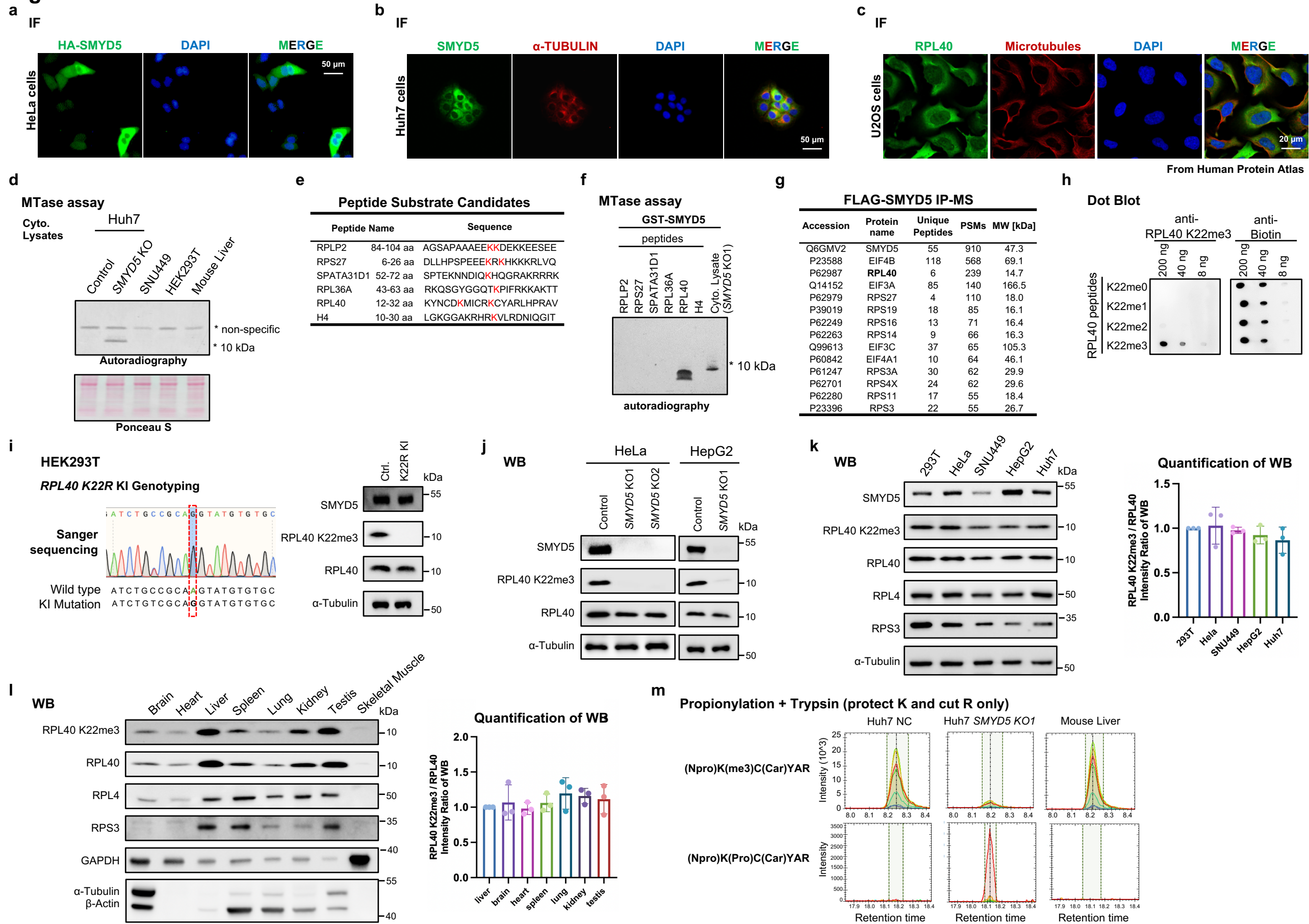

Supplement: Supplementary file 3 — Supplementary information, Fig S3 [file 41422_2024_1013_MOESM3_ESM.pdf]

**Fig. S4**

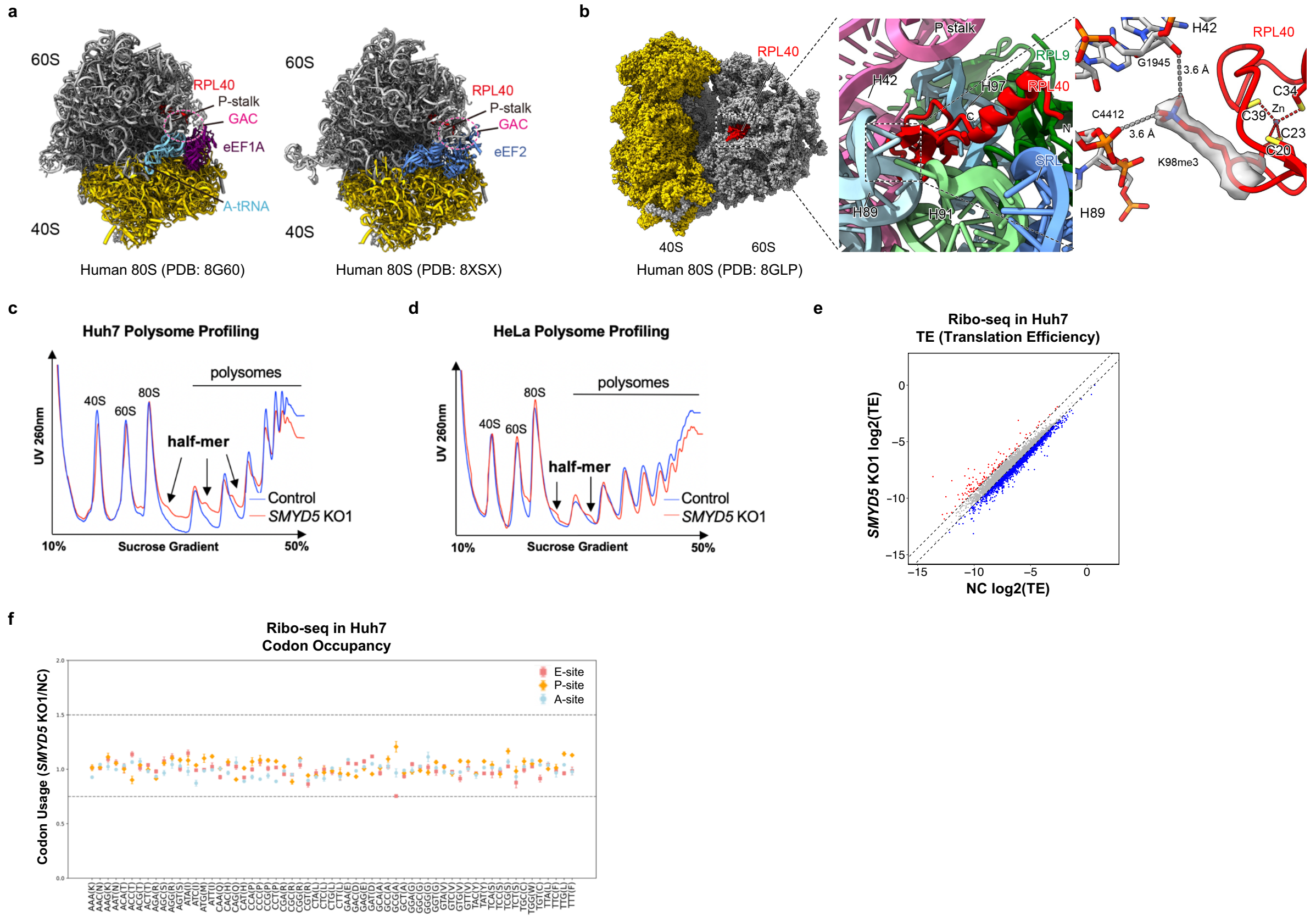

Supplement: Supplementary file 4 — Supplementary information, Fig S4 [file 41422_2024_1013_MOESM4_ESM.pdf]

**a**

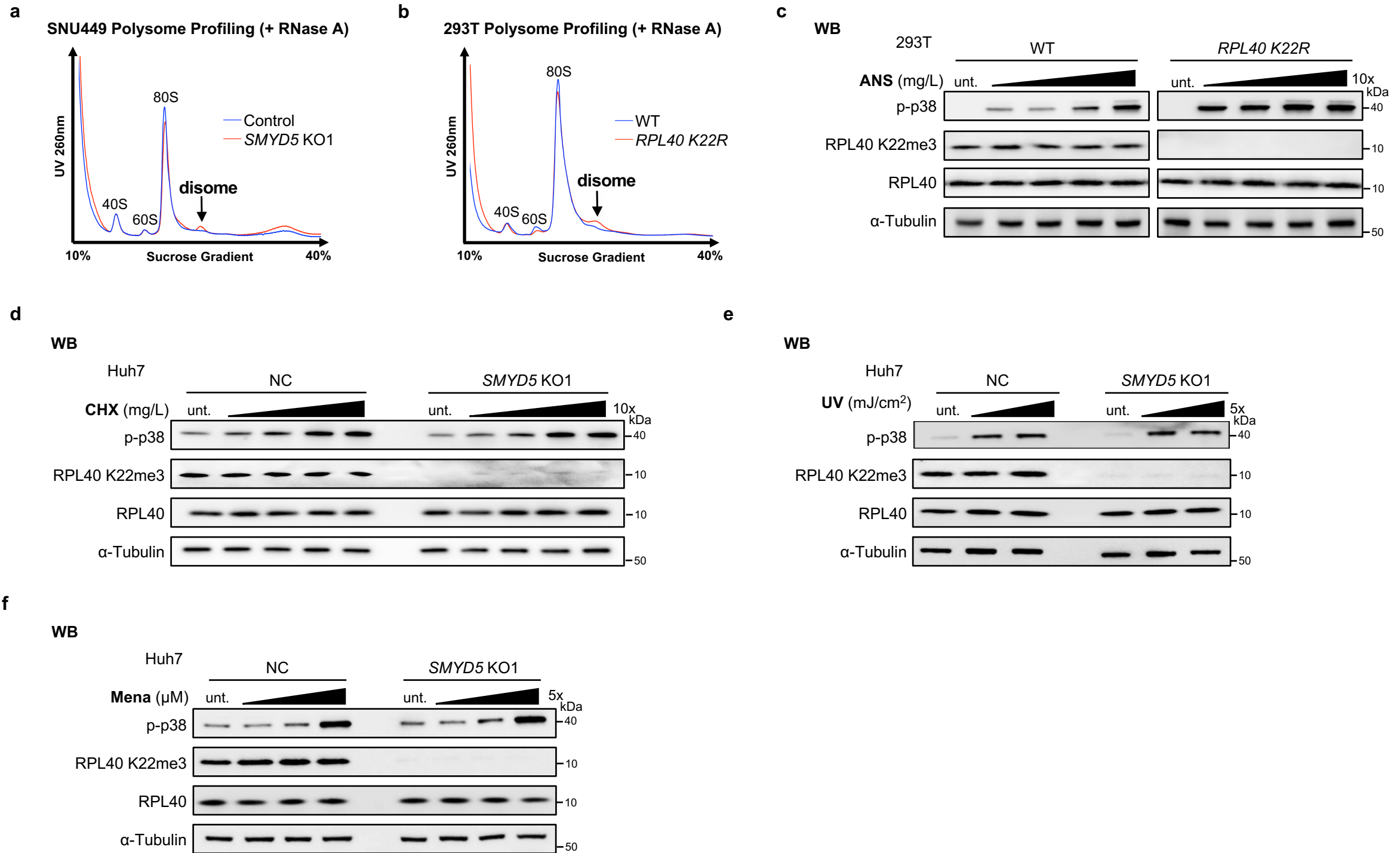

Supplement: Supplementary file 5 — Supplementary information, Fig S5 [file 41422_2024_1013_MOESM5_ESM.pdf]

Fig. S7

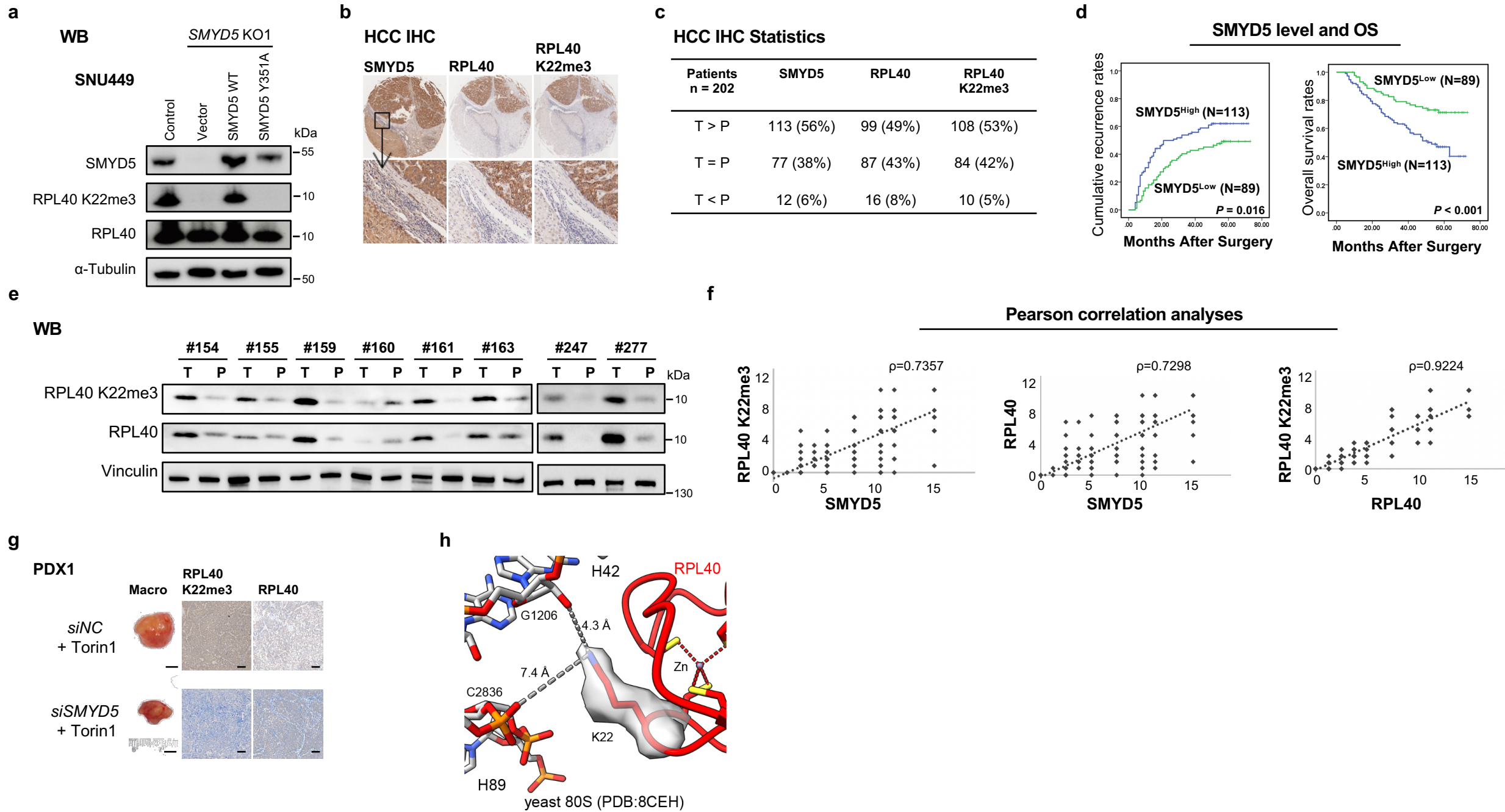

Supplement: Supplementary file 7 — Supplementary information, Fig S7 [file 41422_2024_1013_MOESM7_ESM.pdf]

**Fig. S8**

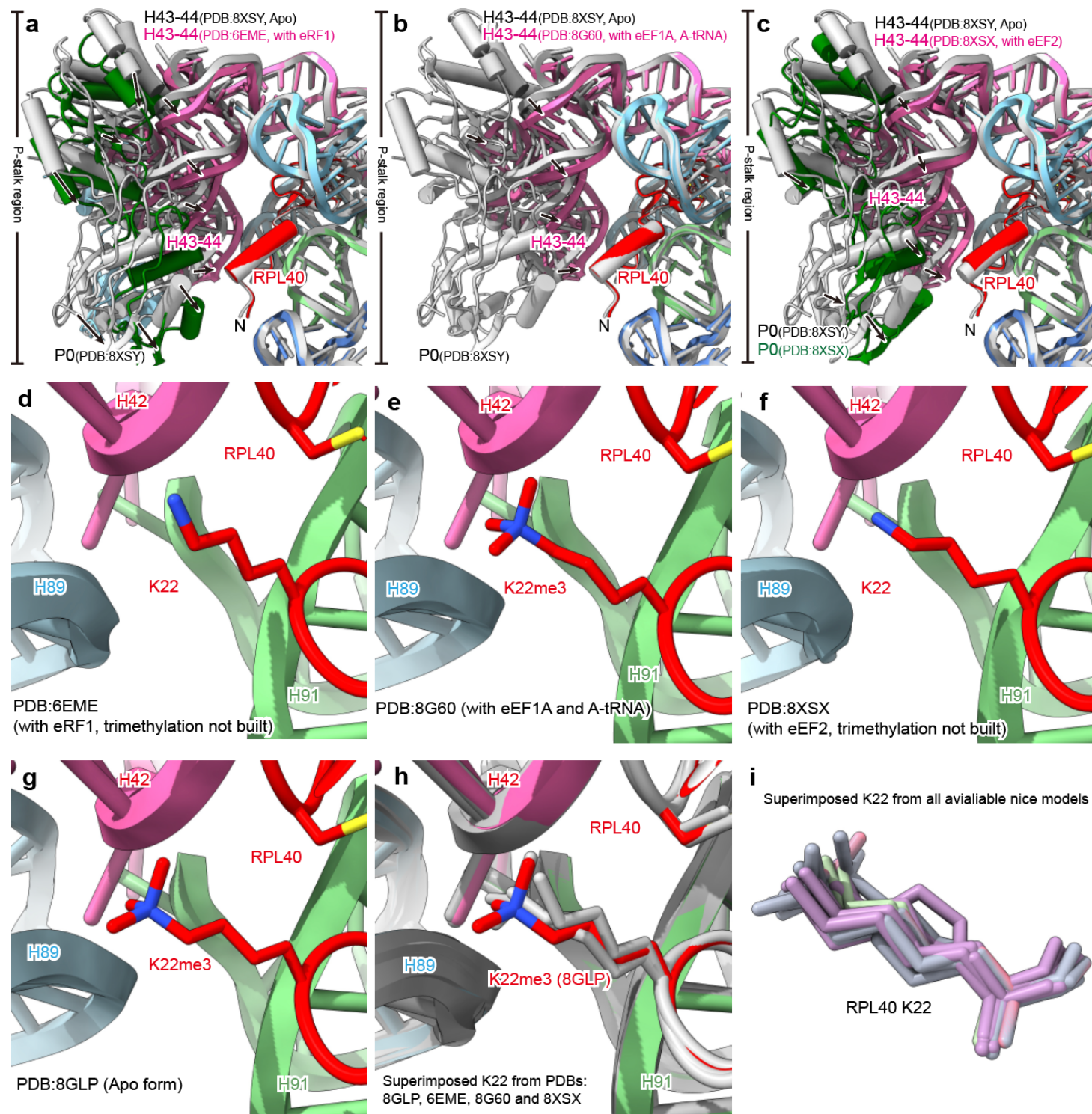

Supplement: Supplementary file 8 — Supplementary information, Fig S8 [file 41422_2024_1013_MOESM8_ESM.pdf]
