# Supplementary material for: SMYD5 is a ribosomal methyltransferase that catalyzes RPL40 lysine methylation to enhance translation output and promote hepatocellular carcinoma
Source: Cell Res. 2024 Aug 5;34(9):648–60. doi: 10.1038/s41422-024-01013-3 (PMC11369092; doi:10.1038/s41422-024-01013-3)

Fig. S2

a

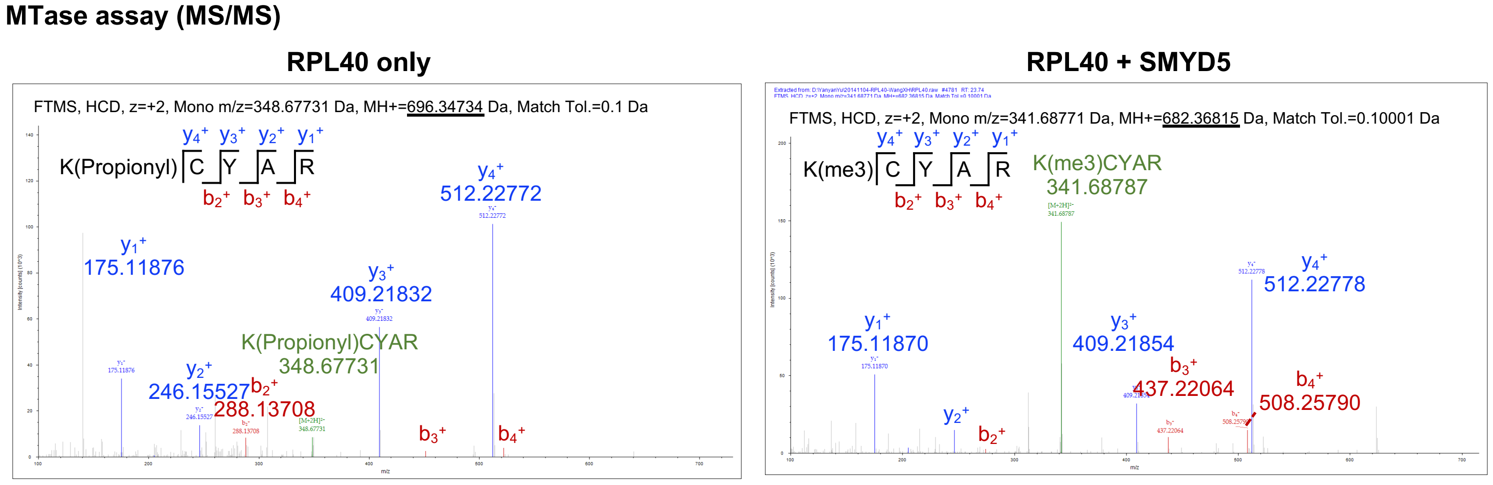

b

MTase assay (MALDI-TOF/TOF)

b+ (m/z)		RPL40 12-32 aa (biotinylated)			y+ (m/z)	
peptide only	peptide +SMYD5				peptide only	peptide +SMYD5
---	---	b1	Y	y20	---	---
504.2	504.2	b2	N	y19	2277.1	2319.2
607.2	607.2	b3	C	y18	2163.1	2205.1
722.2	722.2	b4	D	y17	2060.1	2102.1
850.3	850.3	b5	K	y16	1945.1	1987.1
981.4	981.4	b6	M	y15	1817.0	1859.0
1094.4	1094.4	b7	I	y14	1685.9	1728.0
1197.5	1197.5	b8	C	y13	1572.8	1614.9
1353.6	1353.6	b9	R	y12	1469.8	1511.9
1481.7	1523.7	b10	K/Kme3	y11	1313.7	1355.8
1584.7	1626.7	b11	C	y10	1185.6	1185.6
1747.7	1789.8	b12	Y	y9	1082.6	1082.6
1818.8	1860.8	b13	A	y8	919.6	919.6
1974.9	2016.9	b14	R	y7	848.5	848.5
2087.9	2130.0	b15	L	y6	692.4	692.4
2225.0	2267.1	b16	H	y5	579.3	579.3
2322.1	2364.1	b17	P	y4	442.3	442.3
2478.2	2520.2	b18	R	y3	345.2	345.2
2549.2	2591.2	b19	A	y2	189.1	189.1
---	---	b20	V	y1	118.1	118.1

c

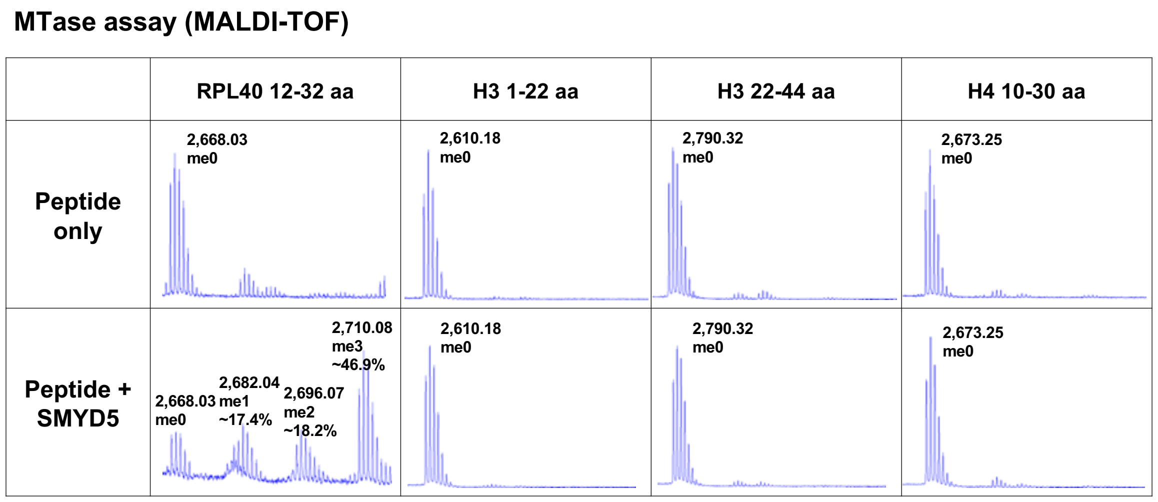

d

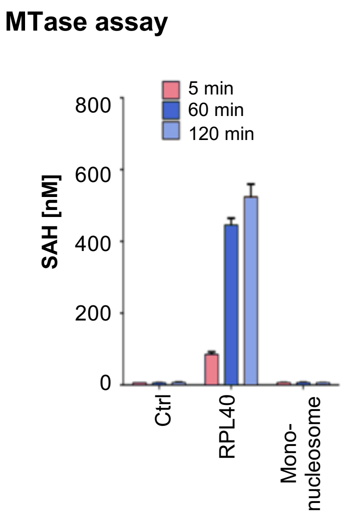

Supplement: Supplementary file 2 — Supplementary information, Fig S2 [file 41422_2024_1013_MOESM2_ESM.pdf]
