# Supplementary material for: SMYD5 is a ribosomal methyltransferase that catalyzes RPL40 lysine methylation to enhance translation output and promote hepatocellular carcinoma
Source: Cell Res. 2024 Aug 5;34(9):648–60. doi: 10.1038/s41422-024-01013-3 (PMC11369092; doi:10.1038/s41422-024-01013-3)

Fig. S6

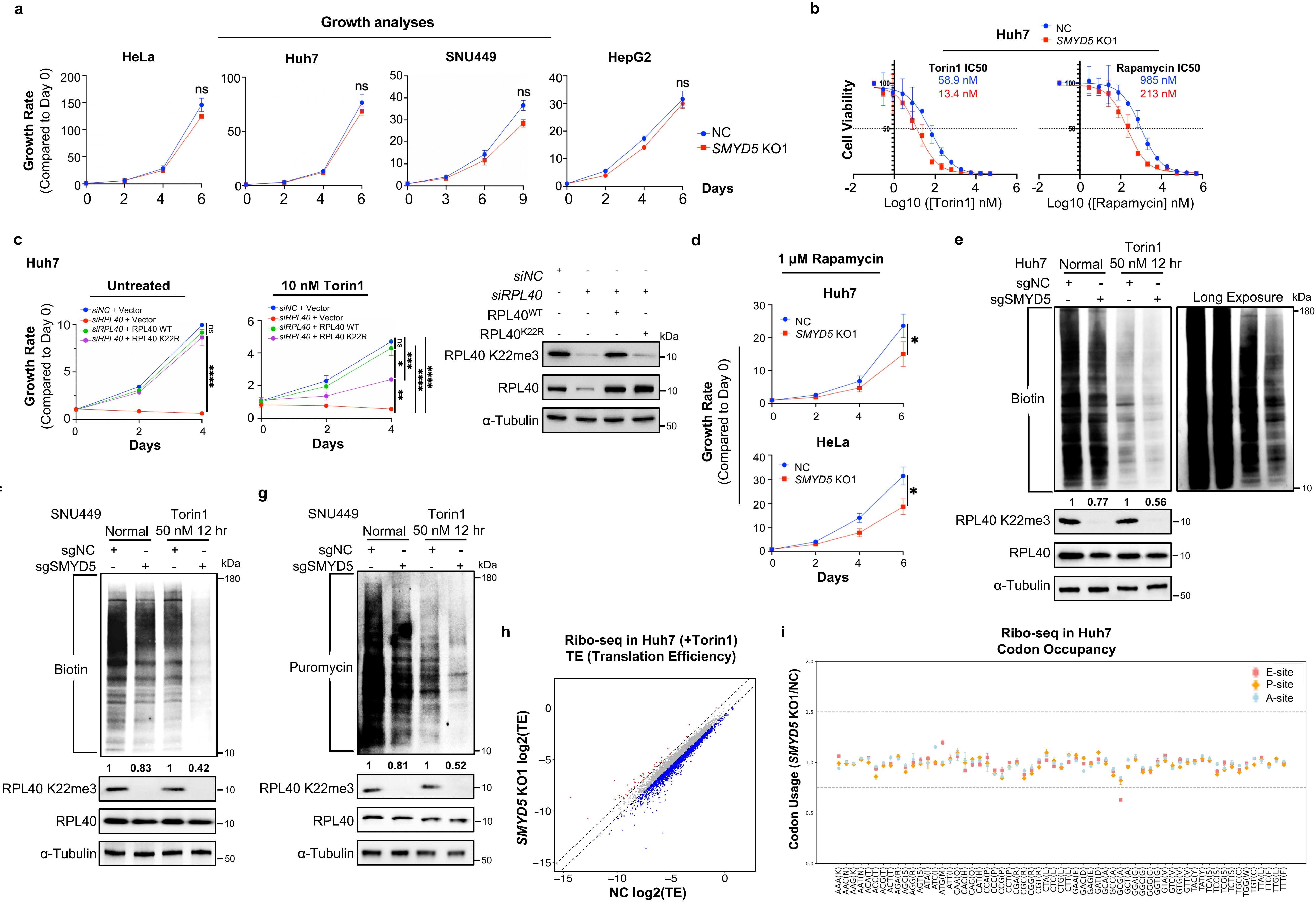
a
Growth analyses
HeLa
Huh7
SNU449
HepG2
Growth Rate (Compared to Day 0)
ns
Days
NC
SMYD5 KO1
b
Huh7
NC
SMYD5 KO1
Cell Viability
Torin1 IC50
58.9 nM
13.4 nM
Rapamycin IC50
985 nM
213 nM
Log10 ([Torin1] nM)
Log10 ([Rapamycin] nM)
c
Huh7
Untreated
10 nM Torin1
siNC + Vector
siRPL40 + Vector
siRPL40 + RPL40 WT
siRPL40 + RPL40 K22R
Growth Rate (Compared to Day 0)
Days
siNC
siRPL40
RPL40WT
RPL40K22R
kDa
RPL40 K22me3
RPL40
α-Tubulin
d
1 μM Rapamycin
Huh7
HeLa
NC
SMYD5 KO1
Growth Rate (Compared to Day 0)
Days
e
Huh7
Normal
Torin1 50 nM 12 hr
sgNC
sgSMYD5
Long Exposure
kDa
Biotin
1
0.77
1
0.56
RPL40 K22me3
RPL40
α-Tubulin
f
SNU449
Normal
Torin1 50 nM 12 hr
sgNC
sgSMYD5
kDa
Biotin
1
0.83
1
0.42
RPL40 K22me3
RPL40
α-Tubulin
g
SNU449
Normal
Torin1 50 nM 12 hr
sgNC
sgSMYD5
kDa
Puromycin
1
0.81
1
0.52
RPL40 K22me3
RPL40
α-Tubulin
h
Ribo-seq in Huh7 (+Torin1)
TE (Translation Efficiency)
SMYD5 KO1 log2(TE)
NC log2(TE)
i
Ribo-seq in Huh7
Codon Occupancy
E-site
P-site
A-site
Codon Usage (SMYD5 KO1/NC)

Fig. S6

j

Gene Signature	NES	NOM *P*-val
HALLMARK_G2M_CHECKPOINT	1.26	<0.001
HALLMARK_MYC_TARGETS_V2	1.25	<0.001
HALLMARK_E2F_TARGETS	1.22	<0.001
GOBP_RIBOSOME_BIOGENESIS	1.20	<0.001
HALLMARK_MYC_TARGETS_V1	1.20	0.096

k

Enrichment plot
GOBP_RIBOSOME_BIOGENESIS

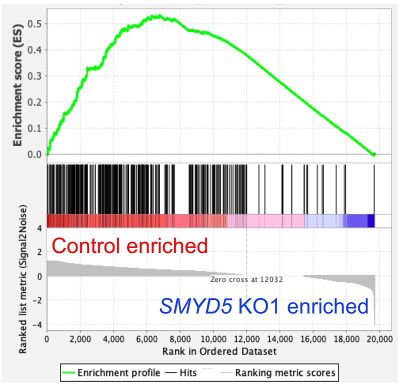

l

Enrichment plot
HALLMARK_MYC_TARGETS V1

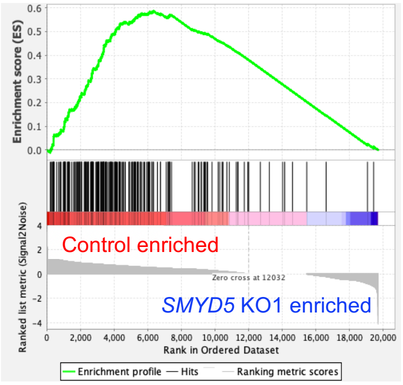

m

Polysome Profiling
with 50 nM Torin1, 12 hr

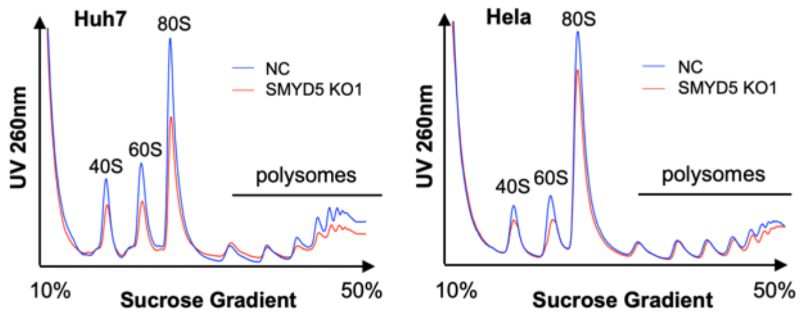

n

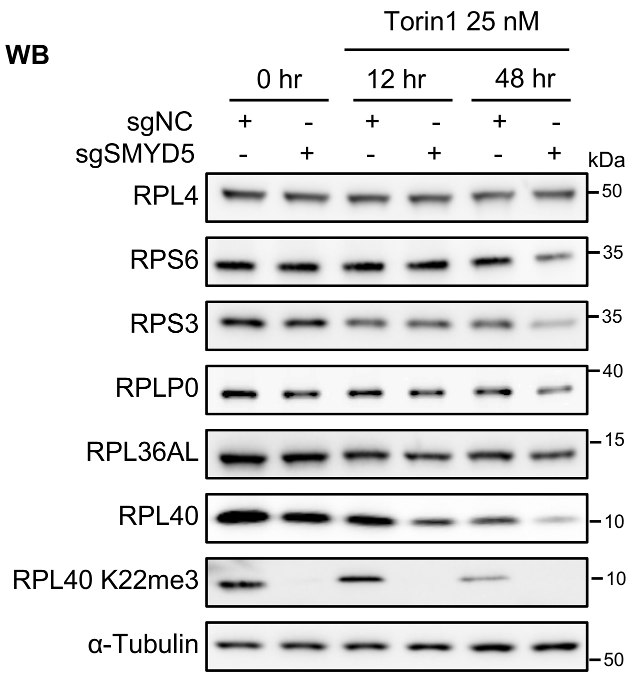

Supplement: Supplementary file 6 — Supplementary information, Fig S6 [file 41422_2024_1013_MOESM6_ESM.pdf]
