# Supplementary material for: SMYD5 is a ribosomal methyltransferase that catalyzes RPL40 lysine methylation to enhance translation output and promote hepatocellular carcinoma
Source: Cell Res. 2024 Aug 5;34(9):648–60. doi: 10.1038/s41422-024-01013-3 (PMC11369092; doi:10.1038/s41422-024-01013-3)
Supplement: Supplementary file 9 — Supplementary information, Figure Legends [file 41422_2024_1013_MOESM9_ESM.pdf]

Figure Legends

Supplementary information, Fig. S1. *SMYD5* mRNA and protein levels are elevated in multiple cancers including HCC.

a TCGA database analyses showed elevated *SMYD5* mRNA levels in the cancer tissues (red) compared to the corresponding normal controls (blue), especially in hepatocellular carcinoma (HCC, in the red dashed box). **b** Strip plot analyses of *SMYD5* mRNA and protein levels in the tumor tissues of HCC samples compared to corresponding paratumor control. Data were retrieved from Qiang Gao, Cell, 2019. **c** Kaplan-Meier's analyses of the overall survival (OS) of patients (n = 160) divided by SMYD5 high and low (mRNA and protein levels, respectively) in HCC. SMYD5 expression levels in HCC were negatively correlated with patients survival with indicated *P* values. Data were retrieved from Qiang Gao, Cell, 2019. **d** Strip plot analyses of SMYD5 protein levels in the tumor tissues of HCC samples compared to corresponding paratumor controls. Data were retrieved from Ying Jiang, Nature, 2019. **e** Kaplan-Meier's analyses of the overall survival (OS) and disease-free survival (DFS) of patients divided by SMYD5 high and low (protein level) in HCC. SMYD5 expression levels in HCC were negatively correlated with OS and DFS with indicated *P* values. Data were retrieved from Ying Jiang, Nature, 2019. Note: All *P*-values above and below were determined by a two-tailed unpaired t-test and $*P < 0.05$, $** P < 0.01$, $*** P < 0.001$, $****P < 0.0001$.

Supplementary information, Fig. S3. Related to Fig. 2.

a-b IF analyses of exogenous HA tagged SMYD5 in HeLa cells and endogenous SMYD5 in Huh7 cells. **c** IF analyses of endogenous UBA52 in U2OS cells from Human Protein Atlas. The images are avaliable from v23.0.proteinatlas.org. URL: https://www.proteinatlas.org/ENSG00000221983UBA52/subcellular **d** In vitro HMT methylation reactions using recombinant GST-SMYD5 and lysates from human cell lines or mouse normal liver as substrates. **e** Potential methylated sequences and the candidate modified lysines were shown in red. **f** Autoradiography analyses of *in vitro* methylation reactions with recombinant SMYD5 using the indicated peptides as substrates. Cytoplasmic protein lysates from *SMYD5* KO1 cells were used as control (last lane). * denoted the candidate substrate signal from cytoplasmic lysate. **g** Top proteins identified by mass spectrometry (MS) in Flag-SMYD5 immunoprecipitation (IP), ranked by the values of peptide-spectrum match (PSM). **h** Specificity test of the RPL40 K22me3 antibody by dot blot using the indicated peptides. All peptides were modified by biotin and the anti-biotin signals were used as loading controls (right). **i** Left, genotyping of *RPL40 K22R* KI HEK293T cell line. Right, WB analyses of SMYD5, RPL40 K22me3 and RPL40 in the control and *RPL40 K22R* KI HEK293T cells. **j** WB analyses of SMYD5, RPL40 K22me3 and RPL40 in the indicated cell lines. *SMYD5* KO1 and KO2 cell lines of HeLa and *SMYD5* KO1 cell line of HepG2 were generated by corresponding gRNAs in Methods. **k-l** WB analyses of SMYD5, RPL40 K22me3, RPL40 and some core ribosome proteins in the indicated cell lines and mouse normal tissues. α-Tubulin, β-Actin or GAPDH were used as control. Intensities of WB were quantified in the right panels, relative ratios were normalized to 293T (**k**) or liver (**l**). **m** PRM ion transitions from methylated or unmethylated K22 precursor peptide by Propionylation + Trypsin strategy (see methods).

Supplementary information, Fig. S4. RPL40 K22me3 structural proximity to 28S rRNA in ribosomal GAC and affect polysome profiles.

a Overview of RPL40 and its lysine 22 (K22) in a ribosome structure. Functional regions such as the P stalk, GAC, L1 stalk, PTC (peptidyl transferase center), CP (central protuberance) are indicated. Translation factors are highlighted in distinct colors. **b** Left, RPL40 in complex with human 80S ribosome; middle, a closer view of RPL40 in complex with 28S rRNA; right, distances between methylated K22 and C4412 of the H89 loop (28S rRNA) and methylated K22 and G1945 of the H42 loop (28S rRNA) are indicated. The analysis of the ribosome structure and its density map is based on PDB 8GLP. K22me3 is marked as "K98me3 of eL40" in the original PDB file. Different helix are highlighted in different colors. **c-d** Polysome profiles of the NC and *SMYD5* KO1 Huh7 (**c**) and HeLa (**d**) cell lines. Black arrows denoted the half-mers. **e** Scatterplots of ribo-seq showing translation efficiency (TE) correlation between NC and *SMYD5* KO1 Huh7 cells. Genes with fold change > 1.5 or < 1/1.5 were colored. **f** Codon bias analysis of ribo-seq between control and *SMYD5* KO1 Huh7 cells. The fold changes of each codon occupancy were calculated, and the dashed lines marked 1.5-fold threshold.

Supplementary information, Fig. S5. Related to Fig. 4.

a-b Polysome profiles from lysates with RNase A treatment of NC and *SMYD5* KO1 SNU449 (**a**) and WT and *RPL40 K22R* KI 293T(**b**) cell lines. Black arrows denoted the disomes. **c** WB analyses for phosphorylation of p38 in the WT and *RPL40 K22R* KI 293T cell lines treated with ANS (0.01-10 mg/L, 15 min). **d-f** WB analyses for phosphorylation of p38 in the NC and *SMYD5* KO1 Huh7 cell lines treated with cycloheximide (CHX) (0.1-100 mg/L, 15 min) (**d**), 254 nm UV irradiation (1-5 mJ/cm^2, recovery 15 min) (**e**) or menadione (Mena) (1-25 μM, 15 min) (**f**) .

Supplementary information, Fig. S6. Related to Fig. 5.

a Proliferation analyses of the control and SMYD5 KO of HeLa, Huh7, SNU449 and HepG2 cells. All data are represented as mean ± SD from three biological replicates. Two-tailed unpaired t-test. **b** Dose-response curves and IC50 measurement of the control and SMYD5 KO1 Huh7 cells treated with Torin1 (left) and Rapamycin (right) for 4 days. Data were represented as mean ± SD, and normalized to the untreated cells. Experiments were performed in duplication. **c** Proliferation analyses of the siNC and siRPL40 Huh7, with the indicated rescuing constructs and under the indicated situation (left and middle). The effect of rescue was verified by WB analyses (right). The proliferation analyses were performed in biological duplication as mean ± SD, *p < 0.05, ** p < 0.01, two-tailed unpaired T-test. **d** Proliferation analyses of the control and SMYD5 depleted (KO1) Huh7 and HeLa cells under the treatment of Rapamycin at the indicated concentrations. Experiments were performed three times. **e** WB analyses of newly synthesized proteins in the NC and *SMYD5* KO1 Huh7 cell lines with or without Torin1 treatment by AHA-click labeling labeling approach. **f-g** WB analyses of newly synthesized proteins in the NC and *SMYD5* KO1 SNU449 cell lines with or without Torin1 treatment by AHA-click labeling (**e**) and puromycin (**f**) labeling approach. **h** Scatterplots of ribo-seq showing translation efficiency (TE) correlation between NC and *SMYD5* KO1 Huh7 cells under Torin1 treatment. Genes with fold change > 1.5 or <1/1.5 were colored. **i** Codon bias analysis of ribo-seq between control and *SMYD5* KO1 Huh7 cells under Torin1 treatment. The fold changes of each codon occupancy were calculated, and the dashed lines marked 1.5-

fold threshold. **j-l** Gene Set Enrichment Analysis (GSEA) identified enrichment of indicated gene sets in ribo-seq. Normalized enrichment scores (NES) and nominal *P* values (NOM *P*-val) were provided. **m** Polysome profilings of the control and SMYD5 depleted (KO1) Huh7 and HeLa cells under 50 nM Torin1 treatment for 12 hours. **n** WB analyses for some ribosome proteins abundance changes during Torin1 treatment in the NC and *SMYD5* KO1 Huh7 cell lines.

Supplementary information, Fig. S7. Related to Fig. 6 and discussion.

a WB analyses of the indicated SMYD5, RPL40 and RPL40 K22me3 levels in the control, SMYD5 depletion and rescued SNU449 cells used for xenograft analyses in Fig. 6c. **b** IHC analyses of SMYD5, HIF1A, RPL40 and RPL40 k22me3 levels in human HCC tumor (T) samples and matched paratumor (P) tissues. Representative IHC views of 4 consecutive slides stained with indicated antibodies were shown in the upper panels; the corresponding enlarged closer views were shown in the lower panels. **c** The comparison of the IHC intensities of SMYD5, RPL40 and RPL40 K22me3 in 202 human HCC samples. **d** Kaplan-Meier's analyses of the recurrency (left) and overall survival (right) of the 202 HCC patients divided by SMYD5 high and low (IHC intensities). **e** WB analyses of RPL40, RPL40 K22me3 and Vinculin (as control) levels in select HCC cases. T stands for tumor sample, and P stands for paired paratumor tissues. **f** Pearson correlation analyses of the IHC intensities. Left, the correlation of SMYD5 vs RPL40 K22me3; Middle, SMYD5 vs RPL40; Right, RPL40 vs RPL40 K22me3. The correlation coefficients (ρ) were indicated. $P < 2.2e\text{-}16$. **g** Representative gross images of PDX and immunohistochemical staining with the indicated antibodies of tumors from the PDX1 *siNC* + Torin1 group and *siSMYD5* + Torin1 group. Scale bars: 5 mm (whole mount) and 100 μm (histology). **h** RPL40 K22 residue is not methylated in yeast 80S ribosome (PDB: 8CEH). Residue K22 is shown in stick with its corresponding density map.

Supplementary information, Fig. S8. Structural comparisons among Apo ribosome and ribosomes complexed with translation factors eEF1A, eEF2, and eRF1

a-c Overviews of RPL40 in the indicated ribosome structures. **d-h** Enlarged views of the RPL40 K22 side chains in the respective ribosome structures. **i** Across all comparisons, the positions of RPL40 and its K22 side chain exhibit no significant alterations, with variations attributable only to differences in model building. PDB numbers are provided for reference, and P stalk helices are highlighted in different colors.
