# Supplementary material for: SMYD5 is a ribosomal methyltransferase that catalyzes RPL40 lysine methylation to enhance translation output and promote hepatocellular carcinoma
Source: Cell Res. 2024 Aug 5;34(9):648–60. doi: 10.1038/s41422-024-01013-3 (PMC11369092; doi:10.1038/s41422-024-01013-3)
Supplement: Supplementary file 10 — Supplementary information, Table S1 [file 41422_2024_1013_MOESM10_ESM.pdf]

Supplementary information, Table S1. Antibodies used in this study.

Name	Source	Identifier	Dilutions
**WB**			
SMYD5	ABclonal	Cat#A6191	1 : 1000
SMYD5	Sigma	Cat#HPA015514	1 : 500
Lamin B1	Proteintech	Cat#66095-1-lg	1 : 5000
α-Tubulin	Proteintech	Cat#66031-1-Ig	1 : 5000
RPL40	Abcam	Cat#ab109227	1 : 10000
RPL40 K22me3	custom generated		1 : 2000
Phospho-p38	Proteintech	Cat#28796-1-AP	1 : 10000
RPL4	Proteintech	Cat#67028-1-lg	1 : 5000
RPS3	Proteintech	Cat#11990-1-AP	1 : 5000
RPS6	Proteintech	Cat#14823-1-AP	1 : 5000
Vinculin	Cell Signaling Technology	Cat#13901	1 : 2000
Flag	Sigma	Cat#F3165	1 : 5000
Streptavidin -HRP	Life Technologies	Cat#434323	1 : 1000
**IF**			
SMYD5	ABclonal	Cat#A6191	1 : 200
HA	Cell Signaling Technology	Cat#3724	1 : 200
DAPI	solarbio	Cat#C0060	1 : 1000
**IP**			

Anti-FLAG M2 affinity gel	Sigma	Cat#A2220	
RPL40	Abcam	Cat#ab109227	1 : 100
SMYD5	ABclonal	Cat#A6191	1 : 100
Normal rabbit IgG	Santa Cruz Biotechnology	Cat#sc-2027	
**IHC**			
SMYD5	ABclonal	Cat#A6191	1 : 250
SMYD5	Sigma	Cat#HPA015514	1 : 100
pH3	Cell Signaling Technology	Cat#9701	1 : 1000
RPL40	Abcam	Cat#ab109227	1 : 500
RPL40 K22me3	custom generated		1 : 2000
