# Supplementary material for: SMYD5 is a ribosomal methyltransferase that catalyzes RPL40 lysine methylation to enhance translation output and promote hepatocellular carcinoma
Source: Cell Res. 2024 Aug 5;34(9):648–60. doi: 10.1038/s41422-024-01013-3 (PMC11369092; doi:10.1038/s41422-024-01013-3)
Supplement: Supplementary file 12 — Supplementary information, Table S3 [file 41422_2024_1013_MOESM12_ESM.pdf]

Supplementary information, Table S3. Compounds used in drug library screen.

Compound Name	Working Concentration	Inhibitory Ratio (*SMYD5* KO1/NC)
Olaparib (AZD2281, Ku-0059436)	200nM	0.911319504
Odanacatib (MK-0822)	200nM	1.031316901
Tanespimycin (17-AAG)	200nM	1.087523552
Anastrozole	200nM	1.087893896
Aprepitant	200nM	1.04457781
Ispinesib (SB-715992)	200nM	0.901058343
Zibotentan (ZD4054)	100nM	1.02918495
GSK429286A	100nM	0.981534395
Adavosertib （MK-1775）	200nM	0.910247007
Ambrisentan	200nM	1.044900009
Ibrutinib (PCI-32765)	200nM	0.9665821
Turofexorate Isopropyl (XL335)	200nM	1.062983581
Nepicastat (SYN-117) HCl	200nM	0.907145659
Crenolanib (CP-868596)	200nM	1.024199874
CHIR-98014	200nM	1.080231542
LDC1267	200nM	0.96411533
Torin 2	50nM	1.035980404
Carfilzomib (PR-171)	50nM	1.030724678

GNF-2	200nM	0.993039962
JNJ-7777120	200nM	1.111280896
Eprosartan Mesylate	200nM	1.013748036
JNK-IN-8	200nM	0.948640249
Rolapitant	200nM	0.977884563
GSK3787	200nM	0.861638195
Rasagiline	200nM	0.913740278
JANEX-1	200nM	1.006499707
VX-702	200nM	0.847257432
BI-4464	200nM	0.828744004
PP2	200nM	0.842773251
GSK2656157	200nM	0.94394401
SGC 0946	200nM	0.992140279
GSK2334470	200nM	0.89811131
AGI-5198	200nM	0.943777222
Bisindolylmaleimide I (GF109203X)	200nM	0.931795451
Alvelestat (AZD9668)	200nM	0.916020796
UNC0642	200nM	0.947224791
AGI-6780	200nM	0.986106111
PFI-2 HCl	200nM	0.904432403
Poziotinib (HM781-36B)	200nM	0.912321086

LDC000067	200nM	0.901922672
FTI 277 HCl	200nM	1.143453816
Nexturastat A	200nM	0.938202191
Santacruzamate A (CAY10683)	200nM	1.054061188
Picropodophyllin (PPP)	200nM	1.23202818
BPTES	200nM	1.062369516
Emricasan	200nM	0.860269874
EPZ020411 2HCl	200nM	1.055030269
SBI-0206965	200nM	0.97055621
Venetoclax (ABT-199, GDC-0199)	200nM	1.141362159
SB366791	200nM	1.044741028
Etomoxir (Na salt)	200nM	0.988544233
Selonsertib (GS-4997)	200nM	0.91920362
T-3775440 HCl	200nM	0.93390312
BAY-876	200nM	1.14585723
GSK'872 (GSK2399872A)	200nM	0.984042747
LY3214996	200nM	1.051760245
Z944	200nM	1.103401039
DMAT	200nM	1.15417303
pm26TGF-β1 peptide (TFA)	200nM	1.12758108
Bemcentinib (R428)	200nM	1.021975702

FF-10101	200nM	1.005815614
ETC-206 (AUM 001)	200nM	1.063205602
GSK467	200nM	1.058399859
TH588	200nM	0.969350794
BX-795	200nM	1.01857296
Cediranib (AZD2171)	200nM	1.029825411
SHP099	200nM	0.990923371
AZD7648	200nM	1.057386037
SMI-4a	200nM	1.174798759
GSK461364	50nM	1.153861447
Pelitinib (EKB-569)	50nM	1.036961433
RS504393	200nM	1.019870705
Omipalisib	50nM	0.655857915
UK-371804 HCl	200nM	1.029559455
AMG-458	200nM	1.096898209
Daidzin	200nM	1.072568194
Finerenone	200nM	1.037885672
TP-064	200nM	0.970002315
AM966	200nM	1.009542205
IRAK4-IN-1	200nM	1.237955859
IOX2	200nM	1.108777258

Canagliflozin	200nM	1.08162081
Palbociclib (PD-0332991) HCl	200nM	0.967082988
bpV (HOpic)	200nM	1.043614958
VTP50469	200nM	1.0506594
WQ 1	200nM	1.044233596
Pitolisant hydrochloride	200nM	1.078089194
Linagliptin	200nM	0.934215605
ZT-12-037-01	200nM	0.857211151
HS-276	200nM	0.933047447
NVP-CGM097	200nM	0.951346876
HG-9-91-01	200nM	1.033892037
ML347	200nM	0.952825987
Alvimopan	200nM	1.067622754
Diphenyleneiodonium chloride (DPI)	200nM	0.811540995
GSK'963	200nM	0.934410954
GSK'547	200nM	1.051551463
SSR128129E	200nM	0.977970886
TED-347	200nM	1.016815573
MLi-2	200nM	1.228831438
T-26c	200nM	1.114705998
Larotrectinib	200nM	1.114025767

PTC-209 HBr	200nM	0.985129147
CITCO	200nM	1.220365609
Tegoprazan	200nM	1.060317393
RO 5028442 (RG7713)	200nM	1.039736072
SC 236	200nM	1.013045427
A-803467	200nM	1.039916991
Torin1	100nM	0.551457552
Trametinib	100nM	0.840932661
Rapamycin	500nM	0.726116714
Alpelisib	50nM	0.826085523
Gefitinib	50nM	0.848285017
ADU-S100	50nM	0.987128941
Cenicriviroc	50nM	0.927603116
3-Bromopyruvic acid	50nM	0.914605572
Galunisertib	50nM	1.103435781
Ipatasertib	50nM	0.933878351
Crizotinib	50nM	0.942497601
WZB117	50nM	0.942497601
Z-DEVD-FMK	50nM	1.040792957
AZD-5069	50nM	0.963056461
GSK269962A	50nM	0.994255745

Stattic	50nM	0.937989569
AZD-7762	50nM	0.958145326
FX-11	50nM	1.028966413
Osimertinib	50nM	1.067055123
Dasatinib	50nM	0.965696766
SB 203580	50nM	0.904752869
TBHQ	50nM	0.894304265
Pirfenidone	50nM	0.915560856
Vemurafenib	50nM	0.945527567
C75	50nM	0.941983355
AT9283	50nM	1.023221366
Honokiol	50nM	0.971931513
CHIR-99021	50nM	1.017841474
KIN1148	50nM	0.925154839
Ruxolitinib	50nM	0.957365982
Wogonin	50nM	0.946882016
Lonidamine	50nM	1.041984023
Tozasertib	50nM	0.988323275
ABT-737	50nM	0.980623809
Mitapivat	50nM	0.974695733
Nifurtimox	50nM	0.925370008

Amlexanox	50nM	1.095645984
Nutlin-3a	50nM	1.062771737
Venetoclax	50nM	0.976717877
Forsythoside B	50nM	0.945893162
FH535	50nM	0.987395881
EMT inhibitor-1	50nM	1.0152316
Dorsomorphin	50nM	0.879552854
Firsocostat	50nM	0.973075521
Birinapant	50nM	0.983162037
SCH772984	50nM	1.085113111
TEPP-46	50nM	1.080685413
BAY 11-7082	50nM	0.936045092
V-9302 (hydrochloride)	50nM	0.961269473
Ripasudil	50nM	1.098428286
Lorlatinib	50nM	0.965577588
Asiaticoside	50nM	0.977396539
Cinacalcet	50nM	0.936203677
Epacadostat	50nM	1.017436959
SB 202190	50nM	1.056197833
SKL2001	50nM	0.883960965
Fatostatin	50nM	0.836804421

PF-06928215	50nM	1.058183257
Staurosporine	50nM	0.579757716
XMU-MP-1	50nM	0.945015897
A 1070722	50nM	0.955976833
Erlotinib	50nM	0.924302698
Sorafenib	50nM	0.87954172
Prexasertib	50nM	0.782503237
