# Supplementary material for: SMYD5 is a ribosomal methyltransferase that catalyzes RPL40 lysine methylation to enhance translation output and promote hepatocellular carcinoma
Source: Cell Res. 2024 Aug 5;34(9):648–60. doi: 10.1038/s41422-024-01013-3 (PMC11369092; doi:10.1038/s41422-024-01013-3)
Supplement: Supplementary file 13 — Supplementary information, Table S4 [file 41422_2024_1013_MOESM13_ESM.pdf]

Smyd5 knockout Mouse Phenotype Summary

**Mouse Phenotypes**	**Male**	**Female**
Mortality/aging	n.s.	n.s.
Growth/size/body region	n.s.	Increased lean body mass
Reproductive system	n.s.	n.s.
Homeostasis/metabolism	n.s.	n.s.
Cardiovascular system	n.s.	n.s.
Digestive/liver/biliary system	n.s.	n.s.
Behavior/neurological system	Abnormal Gait	Abnormal Gait
Renal/urinary system	n.s.	n.s.
Immune system	n.s.	n.s.
Limbs/digits/tail	n.s.	n.s.
Skeleton	n.s.	Abnormal bone mineralization
Integument or pigmentation	n.s.	n.s.
Craniofacial	n.s.	n.s.
Hearing/vestibular/ear	n.s.	n.s.
Endocrine/exocrine gland	n.s.	n.s.
Vision/eye	n.s.	n.s.

Supplementary information, Table S4. Table Summary of *Smyd5* KO mice phenotype recorded on the website of **International Mouse Phenotyping Consortium**. URL: www.mousephenotype.org
